# Investigating the Specificity of the Dehydration and Cyclization Reactions in Engineered Lanthipeptides by Synechococcal SyncM

**DOI:** 10.1021/acssynbio.2c00455

**Published:** 2022-12-15

**Authors:** Patricia Arias-Orozco, Yunhai Yi, Fleur Ruijne, Rubén Cebrián, Oscar P. Kuipers

**Affiliations:** † Department of Molecular Genetics, University of Groningen, Nijenborg 7, 9747 AG Groningen, The Netherlands; ‡ Department of Clinical Microbiology, Instituto de Investigación Biosanitaria, ibs. GRANADA, San Cecilio University Hospital, Av. De la Innovación s/n, 18016 Granada, Spain

**Keywords:** prochlorosins, lanthipeptide bioengineering, *Synechococcus*, lanthipeptide synthase

## Abstract

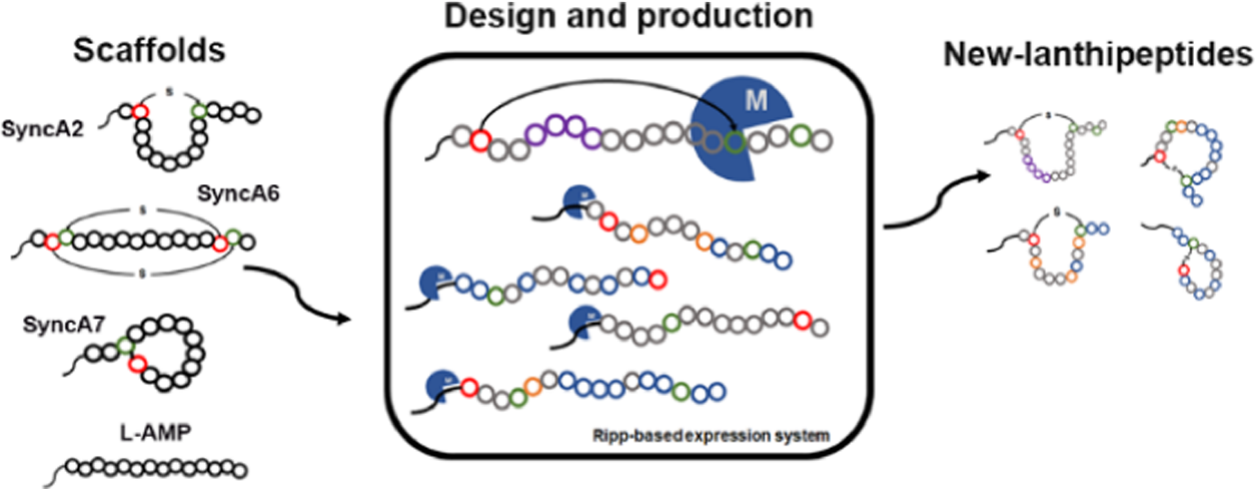

ProcM-like enzymes are class II promiscuous lanthipeptide synthetases that are an attractive tool in synthetic biology for producing lanthipeptides with biotechnological or clinically desired properties. SyncM is a recently described modification enzyme from this family used to develop a versatile expression platform for engineering lanthipeptides. Most remarkably, SyncM can modify up to 79 SyncA substrates in a single strain. Six SyncAs were previously characterized from this pool of substrates. They showed particular characteristics, such as the presence of one or two lanthionine rings, different flanking residues influencing ring formation, and different ring directions, demonstrating the relaxed specificity of SyncM toward its precursor peptides. To gain a deeper understanding of the potential of SyncM as a biosynthetic tool, we further explored the enzyme′s capabilities and limits in dehydration and ring formation. We used different SyncA scaffolds for peptide engineering, including changes in the ring′s directionality (relative position of Ser/Thr to Cys in the peptide) and size. We further aimed to rationally design mimetics of cyclic antimicrobials and introduce macrocycles in prochlorosin-related and nonrelated substrates. This study highlights the largest lanthionine ring with 15 amino acids (ring-forming residues included) described to date. Taking advantage of the amino acid substrate tolerance of SyncM, we designed the first single-SyncA-based antimicrobial. The insights gained from this work will aid future bioengineering studies. Additionally, it broadens SyncM′s application scope for introducing macrocycles in other bioactive molecules.

## Introduction

**Figure 1:**
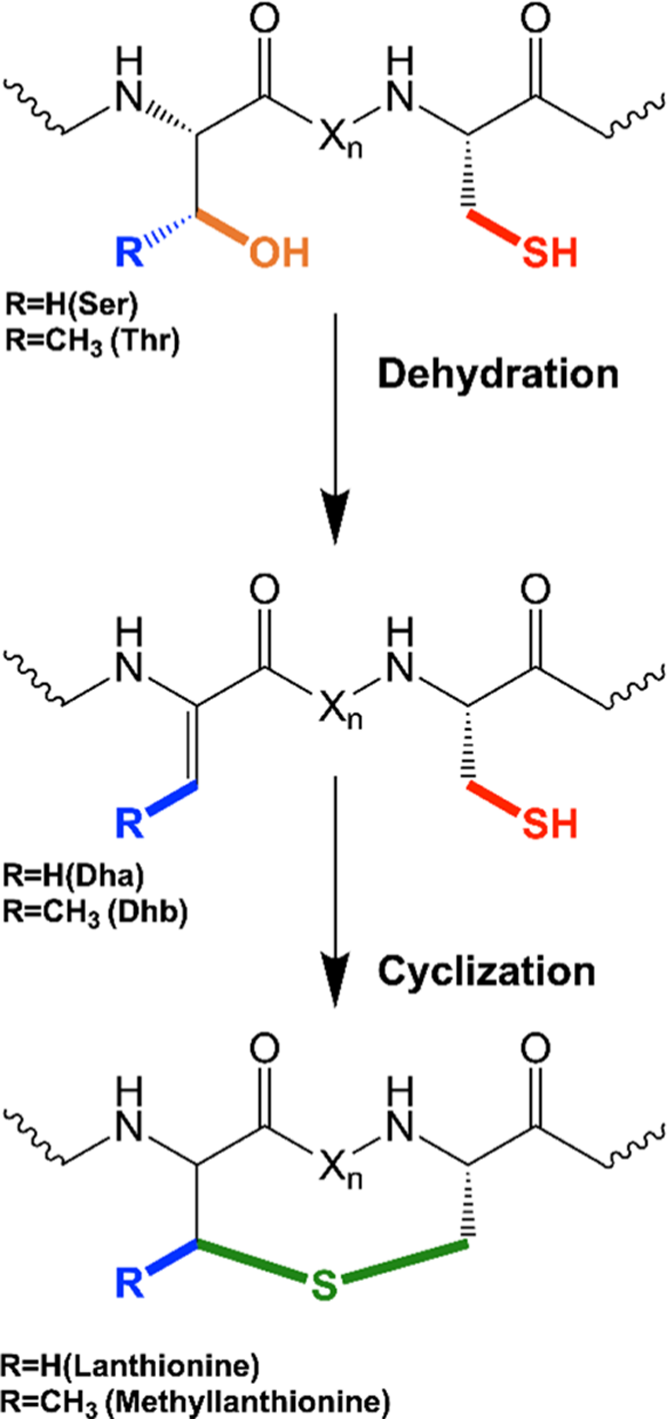
General scheme of the lanthipeptide biosynthetic pathway. Initial dehydration of Ser/Thr residues followed by cyclization through nucleophilic attack of Cys thiol groups and installation of a (β-methyl)lanthionine ring.

Lanthipeptides are ribosomally synthesized and post-translationally modified peptides (RiPPs) with one or more (β-methyl) lanthionine rings.^1^ This family is divided into five classes according to their modification enzymes. In class I, two modification enzymes are found in the biosynthetic gene cluster (BGC), a dehydratase (LanB) and a cyclase (LanC). For class II, both functions (post-translational dehydration and cyclization) are carried out by a bifunctional enzyme (LanM).^1,2^ Finally, class III and class IV enzymes have an N-lyase, a central kinase, and a C-cyclase domain called LanKC or LanL, respectively.^1^ The processing of class II lanthipeptides usually involves a precursor peptide (LanA) with an N-terminal leader peptide recognized by a modification enzyme (LanM) and a C-terminal core peptide that will undergo different catalytic reactions. First, a dehydration step takes place, converting the serine/threonine (Ser/Thr) residues into dehydroalanine and dehydrobutyrine (Dha/Dhb), respectively. This is followed by an intramolecular cyclization step with cysteine (Cys) to form the lanthionine and β-methyllanthionine rings (Figure 1). Finally, the modified peptide is exported, and the leader is cleaved off by a leader peptidase. These steps result in the final product and activation of the core peptide. The latter processes can be performed either by two different enzymes (LanT and LanP) or by one (LanT_p_).^1^

Lanthipeptides form a promising source of stable bioactive compounds with a broad range of biological activities ranging from antimicrobial (lantibiotics), antifungal, or antiviral activities to signaling molecules, channel regulators, and immunomodulatory, antiallodynic, anticonceptive, or anticancer activities.^1,3,4^ Current genome mining methodologies^5,6^ have shown that they are produced by species of all domains of life, with a broad and versatile range of topologies and diverse biosynthetic types of machinery.^7,8^ The extraordinary diversity of modification enzymes and structures of the family of RiPPs provides an attractive synthetic biology platform for combining different modules of enzymes to introduce a variety of post-translational modifications in peptides.^9−11^ For example, it opens up the possibility of synthesizing engineered novel macrocyclic NRPS-like structures without requiring multimodular enzyme complexes for their synthesis.^12,13^ Different biosynthetic machinery have been used to produce lanthipeptides with new structures and biological functions, such as the production of antimicrobials, hormones, protein inhibitors against HIV, and epitope grafting.^13−19^ Moreover, the expression of hybrid peptides with different leaders from RiPP machinery and the development of peptides with enhanced activity and/or stability by the introduction of lanthionine ring(s) have been demonstrated.^15,19−25^ However, some of the modification enzymes used in previous studies display a moderate-to-high substrate specificity, thereby limiting their potential for the modification of unrelated precursor peptides. To overcome this limitation, a new group of lanthipeptide synthetases called ProcM-like enzymes is currently under investigation because of their high substrate promiscuity. This family of broad-range lanthipeptide synthases was found initially in the cyanobacterium *Prochlorococcus* MIT9313, where a single ProcM enzyme can modify 29 precursors named prochlorosins.^26,27^ This peculiar relaxed-substrate-specific feature makes these ProcM-like enzymes and their precursors attractive for bioengineering purposes. Furthermore, it encourages their application in biotechnology to introduce one or more lanthionine rings into non-native peptide precursors.^28^

A new ProcM-like enzyme from *Synechococcus* MIT9509 (SyncM) that catalyzes both dehydration and lanthionine ring formation in lanthipeptides has recently been described.^28^ According to genome mining analysis, this SyncM enzyme can modify the impressive number of 79 different precursors (SyncA) that have very diverse structures.^28^ Six representative SyncA peptides were successfully heterologously expressed and modified in *Lactococcus lactis* by SyncM. The characterized candidates, with either one or two rings, showed different patterns of dehydration and cyclization by SyncM, including the formation of the lanthionine ring in different directions (with respect to the location of Ser/Thr and Cys residues relative to each other). For example, the substrate SyncA6 displays N- to C-terminal ring formation and vice versa. These results demonstrate the broad biosynthetic variability that naturally occurs in this family of RiPPs and the remarkable ability of this enzyme to install a lanthionine ring spanning up to 14 amino acids, including the ring-forming residues.^28^ Macrocyclization of more than 10 residues is a characteristic rarely found in the lanthipeptide family.^29^ Different studies have proposed that macrocyclic structures offer attractive building blocks to improve biological activity, stability, and/or other pharmacological properties of bioactive peptides.^14,30,31^ Furthermore, in the same SyncM study, an in silico analysis suggested a high amino acid tolerance for the flanking amino acids of Ser, Thr, and Cys residues, to enable dehydration and ring formation, thereby diverging from other lanthipeptide synthetases.^28,29^ This tolerance is advantageous for the design of novel lanthipeptides using natural SyncA peptides as templates. However, little is known about the lanthipeptide synthetase′s exact requirements for dehydration and ring formation (e.g., ring size, relative positioning of Ser/Thr and Cys, intertwining of rings, and the number of rings per peptide) and its ability to process nonrelated substrate peptides. It was hypothesized by Arias-Orozco et al. that the peptide preconformation may determine the final processing of the precursor,^28^ as was also mentioned before for ProcM.^1,27^ It is important to note that the biological function of these peptides is still unknown and that antimicrobial activity has thus far not been demonstrated for any prochlorosin or synechococsin (SyncA).

In this study, we have chosen different SyncA substrates as scaffolds for peptide engineering and ring expansion (15-residue lanthionine ring), thereby testing the limits of SyncM in dehydration and circularization. Next, we explored the ability of this enzyme to modify substrates closely related to SyncAs that were rationally designed to harbor the characteristics of known antimicrobial peptides. Finally, we explored the ability of SyncM to install macrocyclic rings in non-lanthipeptide antimicrobials. Overall, we showed the capacity of SyncM to introduce modifications in various substrates and engineered a native nonantimicrobial fully modified and processed SyncA into a biologically active variant. We demonstrate (as a proof of concept) the remarkable flexibility of this expression system, which is very convenient for peptide engineering, particularly for the design of synechococsins with macrocyclic structures, a unique feature of SyncM.

## Results and Discussion

### SyncM Tolerance to Ring Permutations and Size Extension

To gain insights into the specificity of SyncM dehydration and cyclization reactions and to test the flexibility of the proposed expression system, we designed different SyncA derivatives with either one or two rings, using SyncA2 (SA2) and SyncA6 (SA6) as templates, respectively. We selected these two peptides because of their efficient modification, relatively high expression levels, and the presence of a macrocycle. Mutants were coexpressed with pTLR_SyncM in *L. lactis* in a nisin-dual expression system.^28^ Subsequently, purification of expressed mutants was achieved by immobilized metal affinity chromatography (Ni-NTA-IMAC) followed by HPLC. To release the SyncA core peptide, we added an ASPR cleavage site behind the −1 G position, which is conserved in prochlorosin and synechococsin leaders. This ASPR site is recognized by the NisP protease from the nisin biosynthesis machinery. A NisP recognition site was added to all of the leaders used for this study. The dehydration state of the peptides was analyzed by MALDI-TOF MS, followed by N-ethylmaleimide thiol alkylation (NEM),^14,16,28^ to assess lanthionine ring installation. LC-MS/MS measurements were performed on selected peptides to further analyze the lanthionine ring pattern. The results of this section are summarized in Table 1. An estimation of the observed levels of dehydration/cyclization (%) for both sets of mutants is shown in Table 1.

**Table 1:**
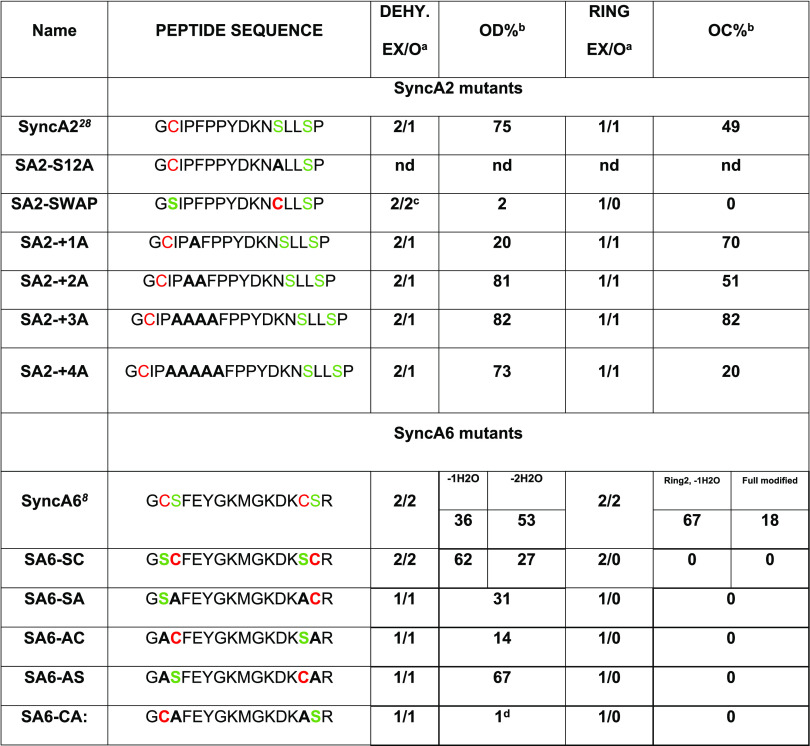
Overview of the Expressed SyncA2 and SyncA6 His6-Tag Mutants Coexpressed with SyncM in *L. lactis* in This Work^e^

a Expected/observed dehydration state and expected/observed cyclization state.

b Percentage values were calculated from the sum of the areas of all possible forms observed in each MALDI-TOF spectrum.

c Low efficiency in dehydration and/or cyclization observed.

d Intensity taken from MALDI spectra linear mode shown in Figure S3C. Blue line.

e Ser/Thr is highlighted in green. Cys is labeled in red. Mutated residues are in bold. OD% and OC% are qualitative estimations of the observed level of dehydration and cyclization in the MALDI-TOF spectra presented in this study, respectively. nd: not determined, MALDI masses not corresponding and/or peptide unstable.

#### SyncM Biosynthetic Tolerance and Limitations for Single-Ring Peptide SyncA2

**Figure 2:**
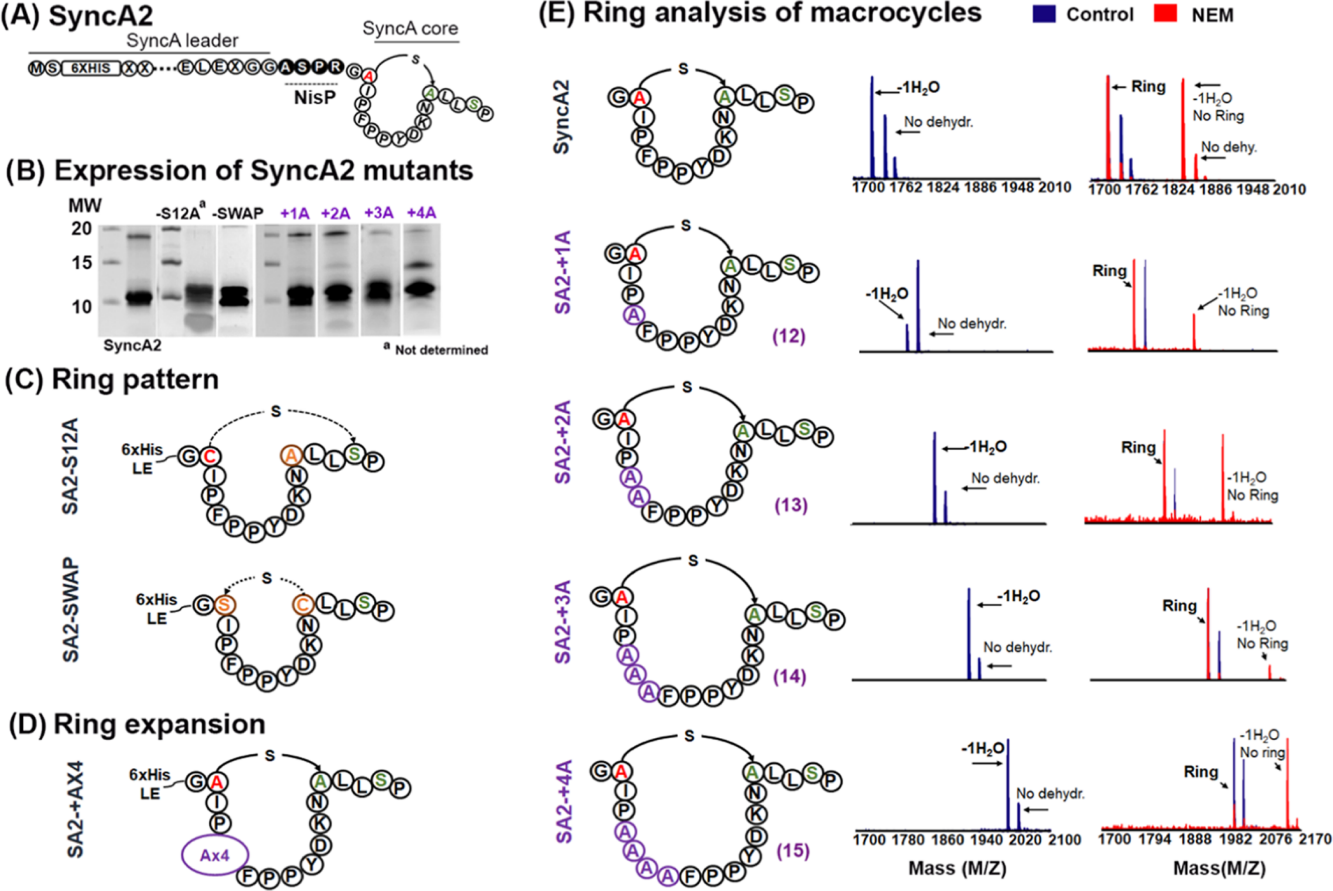
SyncA2 as a scaffold for single-ring formation to assess SyncM flexibility. MALDI-TOF spectra of core peptides after leader removal with NisP protease. (A) General example of the SyncA leader design with His6-tag at the N-terminal and a NisP protease cleave site (ASPR) added after the conserved GG. SyncA core shows the SyncA2 wild-type sequence. The original Cys residue involved in ring formation is depicted in red. Both Ser are depicted in green. (B) Expression of SyncA2 mutants. Ni-NTA His-tag elutions are shown. (C) Schematic representation of SyncA2 variants for investigating Ser selectivity and tolerance to a change in the ring pattern: orange indicates the changed amino acid. (D) Insertion of Ala (in purple) to expand the ring size in the SyncA2 peptide and formation of different sizes of macrocycles. (E) MALDI-TOF MS of the cyclization state of different SyncA2 variants with an increase in the macrocycle size, after NisP cleavage. The number of alanine residues inserted is depicted in purple (including the lanthionine-forming residues). Blue spectra represent the dehydration state. Red spectra represent the outcome of the NEM reaction; ring formation is indicated.

The SyncA2^28^ precursor was selected as a one-ring scaffold for ring engineering to assess to which degree the SyncA2 ring can be expanded (Figure 2A). We generated different SyncA2 variants to explore: (i) the Ser selectivity of SyncM for the ring formation and peptide stability (SA2-S12A), (ii) the effect of interchanging Ser and Cys on the direction of dehydration and ring installation (SA2-SWAP), and (iii) the tolerance to increasing the ring size by introducing additional Ala residues (SA2–+1A to −+4A).

The first set of variants was designed to test whether SyncM could modify the peptide SyncA2 when the ring pattern was changed (mutants SA2-S12A and SA2-SWAP, Figure 2C). Initially, we tested the ability of SyncM to dehydrate Ser15 (not dehydrated in the wild type^28^) in the absence of Ser12 (mutant SA2-S12A, 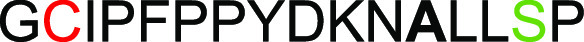) to form a 14-amino-acid ring with Cys2. Even though peptide expression was visible in the tricine-SDS gel, we did not observe the expected masses in the MALDI-TOF spectra (Figure 2A—S12A). Thus, we suspect that SyncM can form no alternative ring with Ser15. Next, we swapped the residues forming the lanthionine single ring, SA2-SWAP (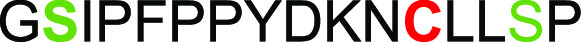), to investigate whether the direction of cyclization could be changed. A good yield and no degradation were achieved at the beginning of the purification (Figure 2B—SWAP). Nevertheless, the mass spectra showed that most of the peptide was nondehydrated, and only a very low amount of dehydrated peptides was observed (Figure S1A). Further, no ring installation was observed (Figure S1A). These data were confirmed by LC-MS/MS (Figure S1B–D). Unexpectedly, fragmentation analysis of this mutant (Figure S1D) revealed that SyncM dehydrated either Ser2 or Ser15. As mentioned before, dehydration of Ser15 is not present in the fully modified and processed SyncA2 wild type. A probable explanation is that the mutation could have altered the preorganization/folding of the peptide, exposing this Ser to the SyncM dehydration domain and highlighting the importance of the prepeptide structure for its processing.^27,28,32^ In conclusion, these data suggest that the preconformation of this peptide is critical for the dehydration profile and shows a strict C- to N- directionality (with respect to the location of Ser/Thr and Cys residues).

We then explored the ability of SyncM to introduce larger macrocycles in variants of SyncA substrates (Figure 2D). For this, we systematically increased the ring size from the original 11 to 15 amino acids by adding Ala residues in front of Phe5 (Figure 2D,E). The introduction of macrocycles in regular peptide scaffolds is an interesting engineering step that can improve bioactivity and maximize interaction with the target protein.^14,30^ All peptides with these macrocycles were expressed (Figure 2A—+1A, +2A, +3A, and +4A). In Figure 2E, the mass spectra show the expected dehydration for all ring expansion mutants in the range of 20–80%D (Table 1). Strikingly, NEM experiments confirmed ring formation in all mutants. Noteworthy, the largest mutant (SA2+4), encompasses a 15-amino-acid ring (including the lanthionine). However, we observed a less efficient ring formation in this mutant (Figure 2E—SA2+4). LC-MS/MS supported these observations (Figure S2). Fragmentation ions corresponding to the noncyclized form of SA2+4A were clearly observed, suggesting less effective ring formation than the smaller ring size mutants (Figure S2B—SA2+4).

In summary, we demonstrate the installation of a 15-amino-acid-long lanthipeptide ring, the largest ring installed by ProcM-like enzymes characterized until now.^28^ The results suggest that, with certain limitations, SyncM could be used as an enzyme for the introduction of a macrocycle in regular peptides, opening the way for the production of NRPS mimics.

#### SyncM Biosynthetic Limitations for SyncA-Based Peptides with Two Rings

**Figure 3:**
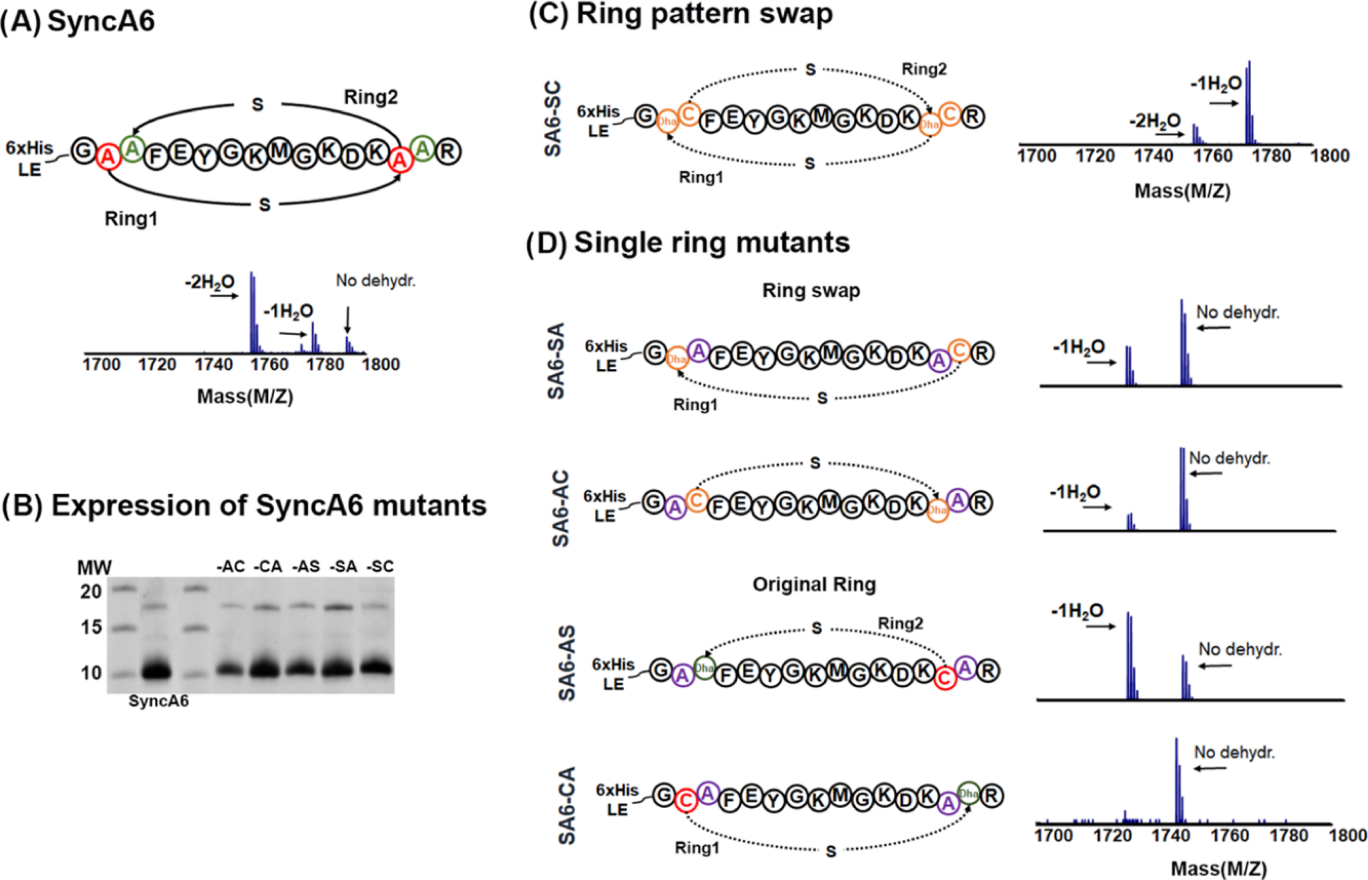
SyncA6 as a scaffold for single-ring formation to evaluate SyncM tolerability. Schematic representation of SyncA6 variants and their MALDI-TOF MS analysis: (A) SyncA6-wt. Original Cys residues involved in ring formation are depicted in red and Ser in green. (B) Expression of SyncA6 mutants: Ni-NTA His-tag elutions are shown. (C) Change in the ring pattern. (D) Alanine substitutions of the lanthionine-forming Cys and Ser residues to determine the ability of SyncM to form single rings in a SyncA6 scaffold. Two subsections: a single ring with a swap of Ser and Cys and the original ring-swapped amino acid are in orange, while substituted Ala is in purple.

To continue exploring SyncM tolerability, we next evaluated the system flexibility in a two-ring peptide using SyncA6 as a model. SyncA6 (Figure 3A) is a unique peptide with an overlapping ring pattern, with the complexity that the installment of macrocycles is in both directions. Similar to the SyncA2 variants, we designed a set of mutants that included a change in ring directionality and used alanine mutagenesis for single-ring formation. All mutants in this subsection were successfully purified (Figure 3B). Dehydration levels varied in all designs (Figure 3C,D and Table 1). However, no rings were formed in any of the variants (Figure S3).

Initially, we aimed to answer whether SyncM has a fixed preference in directionality for dehydration and ring installation. For this purpose, we switched the direction of the ring formation, SyncA6-SC (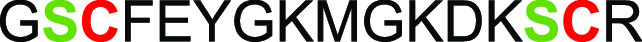, swap in both rings; Figure 3C). Dehydration was present, although with less efficiency than that in the wild-type SyncA6. Unlike SyncA6 (Figure 3C), the one-time dehydrated (−1H_2_O) form is the main peptide population. This contrasts with what was previously found in a modification study with ProcM and Prochlorosin 2.8. In this nonoverlapping two-ringed peptide, the directionality change was accepted for one ring, suggesting topological specificities for ring formation.^16^

Moreover, we designed single-ring mutants for the peptide SyncA6, thereby maintaining the original order of Cys-Ser or reversing it (Figure 3C,D). For the reverse single-ring peptides, SA6-SA (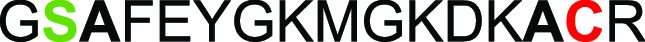) and SA6-AC, (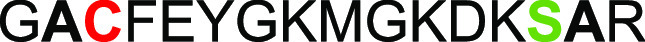), the dehydration profile was less efficient (Figure 3C—SA6-SA and SA6-AC) as compared to both SA6-SC and the wild type (Figure 3A). For the single-ring formation peptides with Ser/Cys in the original order, SA6-AS (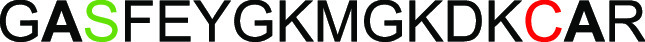, the internal ring) was dehydrated with the highest efficiency (Figure 3D). This observation is in contrast to the mutant SA6-CA 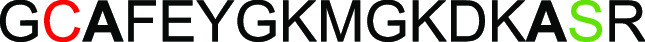 (the external ring), in which almost no dehydration was observed (Figure 3D). The data show that SyncM can dehydrate SyncA6 peptides with several Ser–Cys exchanges, albeit with a cost in efficiency, while there is a strict preference in the cyclization order. This rigid characteristic was also observed for Prochlorosin 1.1.^33^ To conclude, SyncA6 has a critical CS–CS directionality essential to efficient processing.

Together, these results provide important insights into the limitations of the broadness of SyncM′s relaxed-substrate specificity, case-specific to SyncA2 and SyncA6.

### SyncM as a Biosynthetic Tool for Engineering Single-Ringed Synechococsins: Mimicking Cationic Antimicrobial Peptides

In addition to the evaluation of the flexibility of SyncM in ring formation of two different SyncAs,^28^ we proposed that this SyncM expression system could possibly be applied to produce engineered bioactive new-to-nature lanthipeptides. Hence, as a proof of concept, our next step in this study was to determine whether a single-ring SyncA can be used as a scaffold to rationally designed cyclic antimicrobial peptide (cAP) mimics and switch the unknown biological activity of a fully modified and processed SyncA to an antimicrobial activity (Table 2). We selected single-ringed SyncAs based on three criteria: (i) more efficient cyclization for single-ringed SyncAs,^28^ (ii) straightforwardness in ring analysis (fewer intermediates), and (iii) and the observation of single-ring topologies in bioactive peptides (like in many NRPSs). For the rational design of the SyncA-engineered peptides into antimicrobial peptides, known design principles described in the literature were applied,^34,35^ such as amphiphilicity, presence of cationic amino acids, such as Lys and Arg (cationic antimicrobial peptides feature), best amino acid profile flanking Ser/Thr,^28,29^ and the use of antimicrobial sequence databases.^36,37^

**Table 2:**
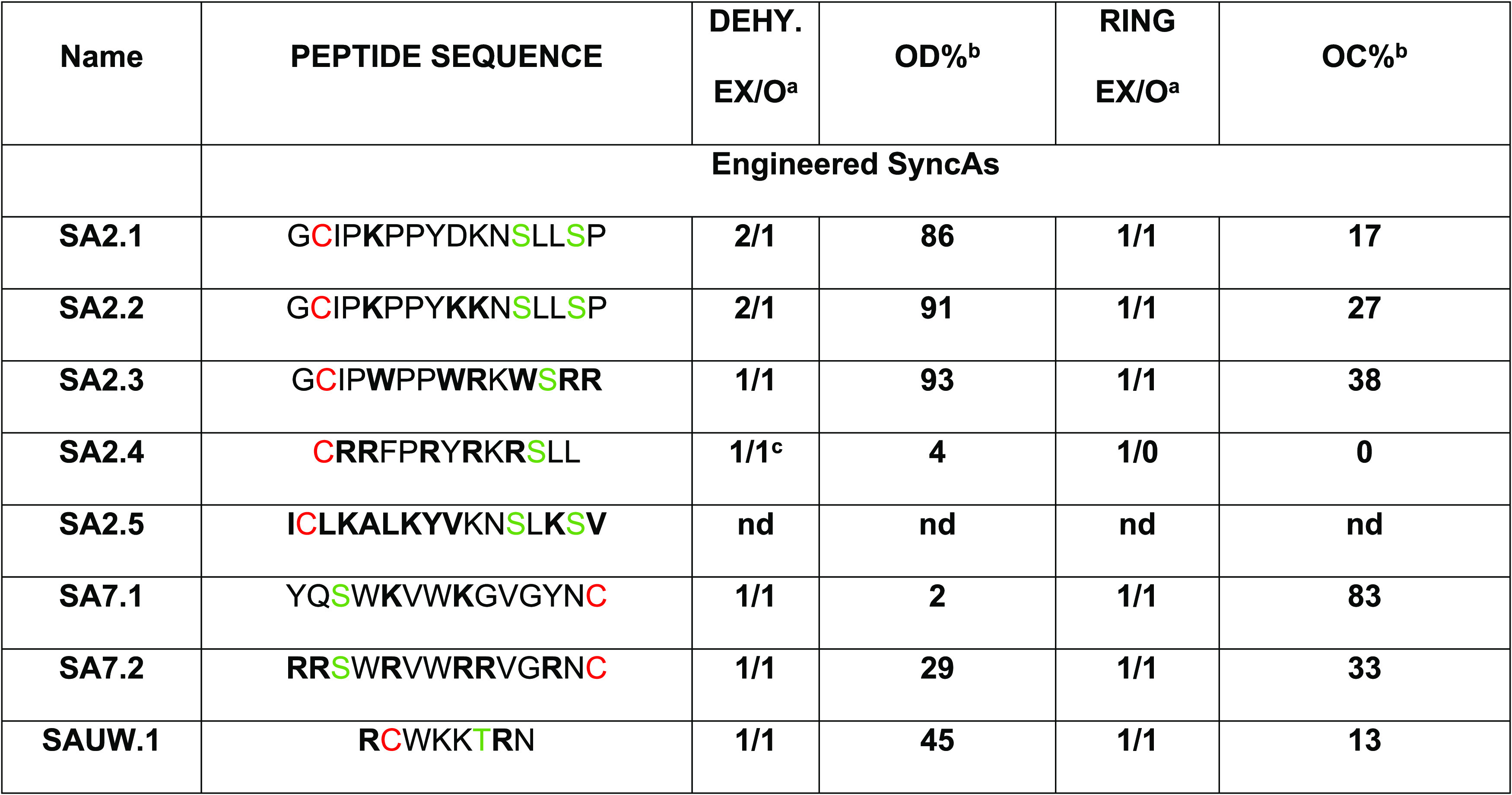
Overview of Expressed (His6-Tag) SyncA Designs Coexpressed with SyncM in *L. lactis* in This Work^d^

a Expected/observed dehydration state and expected/observed cyclization state.

b Percentage values were calculated from the sum of the areas of all possible forms observed in each MALDI-TOF spectrum.

c Low efficiency in dehydration and/or cyclization observed.

d Ser/Thr is highlighted in green. Cys is labeled in red. Mutated residues are in bold. OD% and OC% are the qualitative estimations of the observed level of dehydration and cyclization in the MALDI-TOF spectra presented in this study, respectively. nd: not determined, MALDI masses not corresponding and/or peptide unstable.

**Figure 4:**
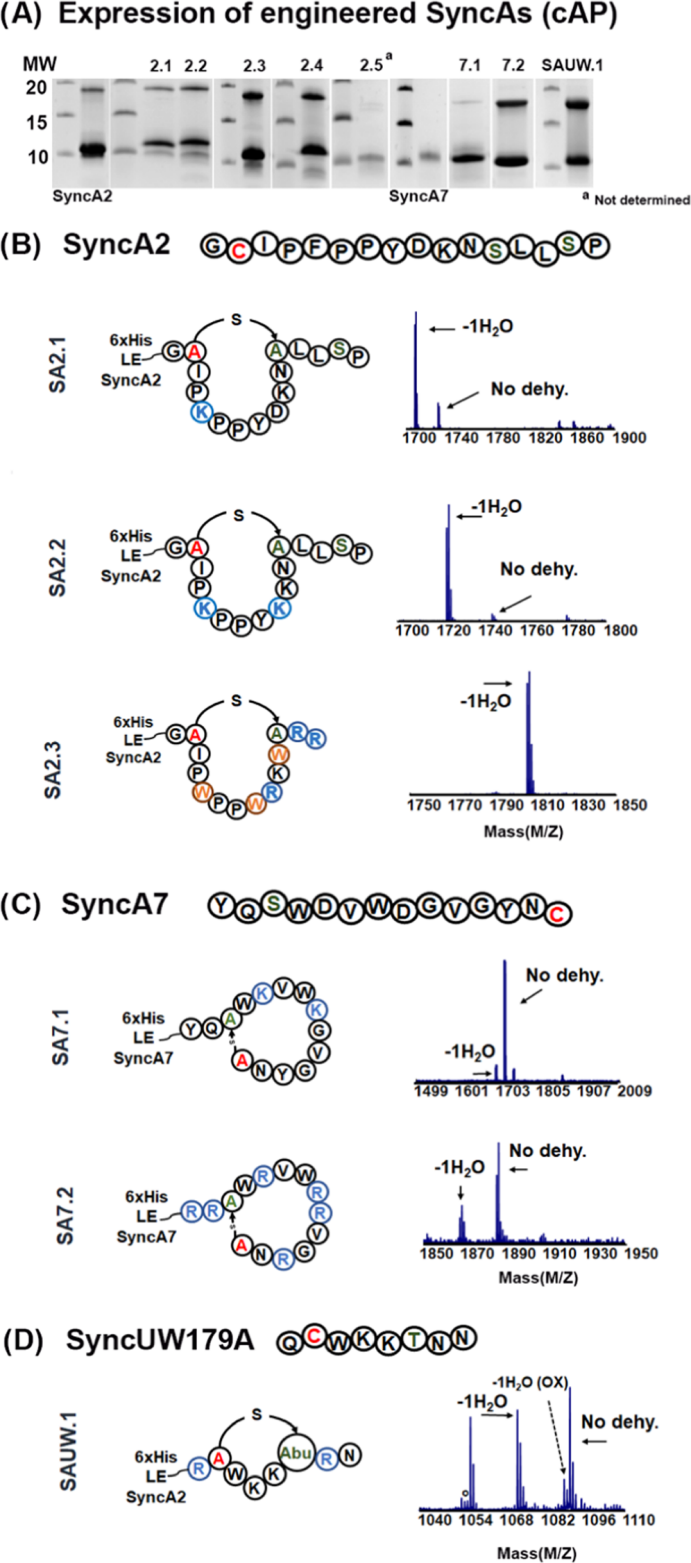
Successfully engineered SyncAs modified by SyncM. MALDI-TOF spectra show the state of dehydration, after the removal of the leader peptide with the NisP protease. (A) Expression of cAP designs after Ni-NTA His-tag purification. First elutions are shown. (B) SyncA2 was used as a template for the design of SyncA2.1, SyncA2.2, and SyncA2.3. Substitution by Trp is depicted in orange, and positively charged amino acids are indicated in blue. (C) SyncA7 was used as a template for the design of mutants SyncA7.1 and SyncA7.2. (D) SyncAUW.1. The template for this cAP design is a synechococsin from the *Synechococcus* UW179A strain.

We cloned the newly designed core peptides with the corresponding leader peptide from which the mutants were derived, with the exception of SAUW.1 (Table 2 and Figure 4). This last variant was derived from *Synechococcus* UW179A. The SAUW.1 wild-type peptide was selected due to the presence of positively charged and hydrophobic amino acids and to test if SyncM would modify a SyncA substrate from a different strain. We maintained a NisP protease cleavage site (ASPR) in the derived peptide from SyncA2 and SAUW.1 to release the core peptide. The SyncA7-derived peptides were cleaved with the LahT_150_^38^ protease, as it was previously reported that fully modified SyncA7 is resistant to NisP cleavage.^28^ Final constructs were coexpressed with SyncM. All mutants were purified efficiently, but less for SyncA2.5 (Figure 4A). Complete modification occurred in almost all peptides, except in SA2.4 (Figure S4A) and SA2.5. The observed levels of dehydration and cyclization from the MALDI-TOF spectra of designed peptides are presented in Figure 4 and Table 2. NEM reactions that further support LC-MS/MS ring analysis can be found in Figure S4B.

#### SyncA2 as a Scaffold for Cyclic Antimicrobial Peptide Mimics

Initially, we hypothesized that these nonantimicrobial SyncA native peptides could be engineered into antimicrobial peptides by replacing the negatively charged or neutral amino acids with positively charged amino acids, as AMPs are frequently cationic. Cationic peptides are more likely to interact with the negatively charged bacterial membrane. A detailed amino acid analysis revealed a fairly even distribution of negative and positive charge residues (Table S1). Moreover, of the putative 79 core peptides of the different SyncAs, 43% have a negative charge, 20% are neutral, and 37% have a positive charge (Table S1).

**Figure 5:**
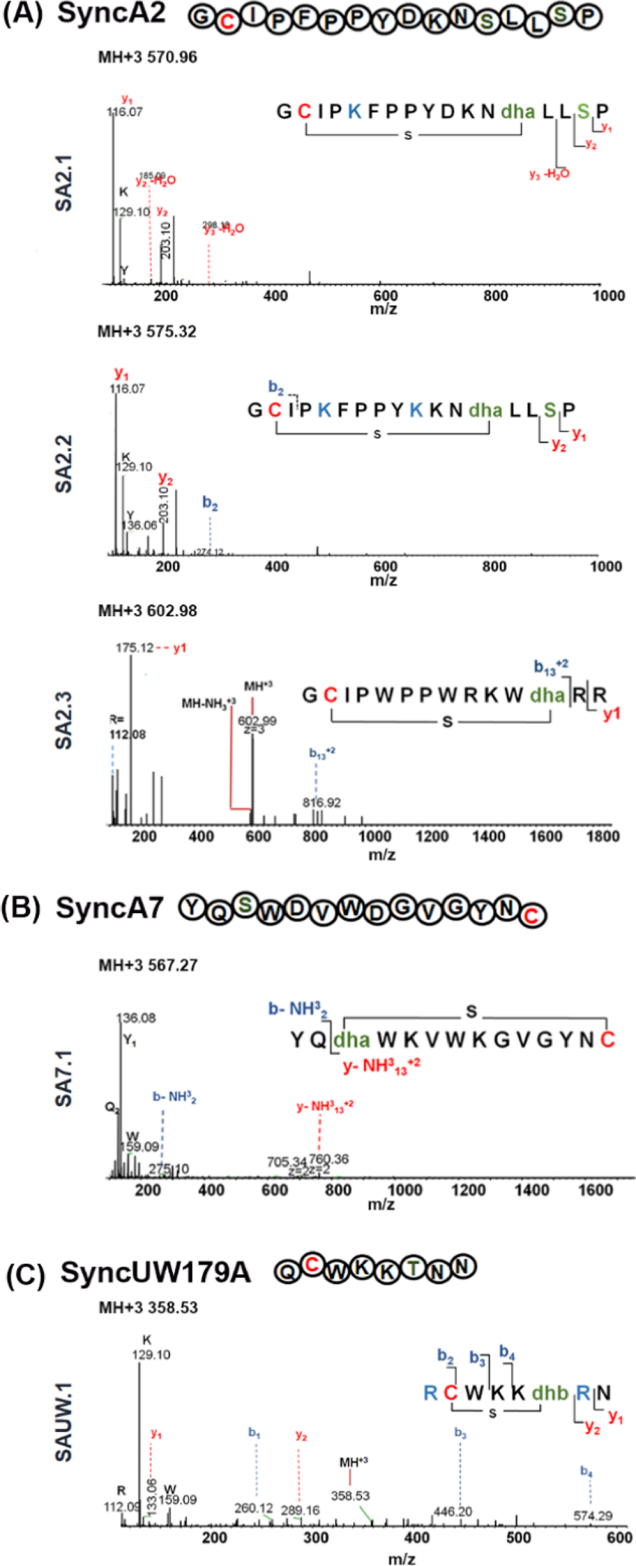
LC-MS/MS analysis of ring formation in engineered SyncAs (cAP) by SyncM. Insights into the dehydration and lanthionine ring pattern are indicated by a black line. Dehydrated Ser/Thr is depicted in green and Cys is in red. (A) SyncA2 designs: SA2.1, SA2.2, and SA2.3; (B) SyncA7 designs: SyncA7.1; (C) SyncAUW179A design: SyncAUW.1.

In this context, the first two variants of SyncA2 (Figure 4B—SA2.1 and SA2.2) were designed to explore the effect of the increase of the positive charge in this scaffold on the antimicrobial activity and modification efficiency by SyncM. In the SA2.1 mutant (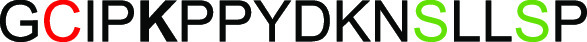), Phe5 was changed to Lys. Prolines were not altered to avoid the risk of losing any potential preformed conformational organization that they could confer. In SA2.2 (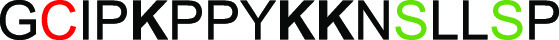), we further substituted D9K, yielding a positively charged peptide (3+). Both variants were expressed at high yields (Figure 4A—SA2.1 and SA2.1), dehydrated (Figures 4B and S5A,B), and cyclized (Figure 5A). Their efficient modification showed that SyncM is tolerant to the substitution of positively charged residues on these positions. Alternatively, hydrophobic residues can penetrate the target bacterial membrane and create a disruption.^35,39^ An example of this is the mutant SA2.3 (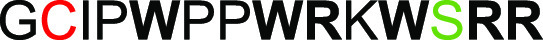). We took advantage of the presence of Pro residues in SyncA2 and introduced Trp and Arg residues to create a mutant displaying similar characteristics as tryptophan- and proline-rich antimicrobial peptides.^40^ This mutant was fully modified (Figure 4B—SA2.3). The expected ring installation can be observed in the fragmentation spectra (Figures 5A and S5C).

In contrast to the results described above, mutant SA2.4 (CRRFPRYRKRSLL) was expressed but not modified, as shown in Figure S4A. In contrast, in SA2.5 (**I**C**LKALKYV**KNSL**K**S**V**), the expression efficiency was low (Figure 4A and Table 2). Thus, we could not determine the correct masses in MALDI-TOF (data not shown). The difference in SyncA2 mutants’ expression and modification is intriguing. We hypothesized that for SyncA2, the constrained backbones of Pro (Pro4, Pro6, and Pro7) might provide a prefolding conformation that facilitates dehydration and cyclization. In a similar case, ProcA2.8, a substrate from ProcM, contains a rigid linker region (Met10–Pro11–Pro12) that could play a role in the preorganization of the linear peptide and promote the formation of its rings.^33^ In a later fragmentation study to analyze the structural signature of ProcA2.8, they observed that substitution of Pro in the core peptide does not affect the ring pattern, although it does alter the environment of the residues in close proximity, resulting in different conformations.^41^

#### Exploring Other Single-Ringed SyncAs as a Scaffold for Cyclic Antimicrobial Mimics

We evaluated two more candidates to assess whether the introduction of positively charged amino acids can also be applied to different single-ring SyncA scaffolds. SyncA7 (Figure 4C) was chosen because of its hydrophobic residues, including Trp^39^ and SyncAUW.1 (Figure 4D) from the *Synechococcus* UW179A. All three designed peptides, SA7.1 (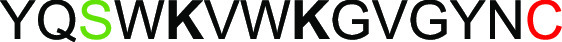), SA7.2 (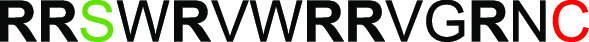), and SAUW.1 (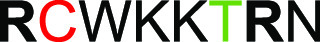), were partially dehydrated (Figure 4C,D). We confirmed ring installation by LC-MS/MS (Figure 5B,C). For mutant SA7.2 (RRSWRVWRRVGRNC), NEM analysis (Figures S4B—SA7.2) suggests actual ring formation. With this variant, difficulties in the downstream processing precluded LC-MS/MS analysis.

#### Antimicrobial Assay

**Figure 6:**
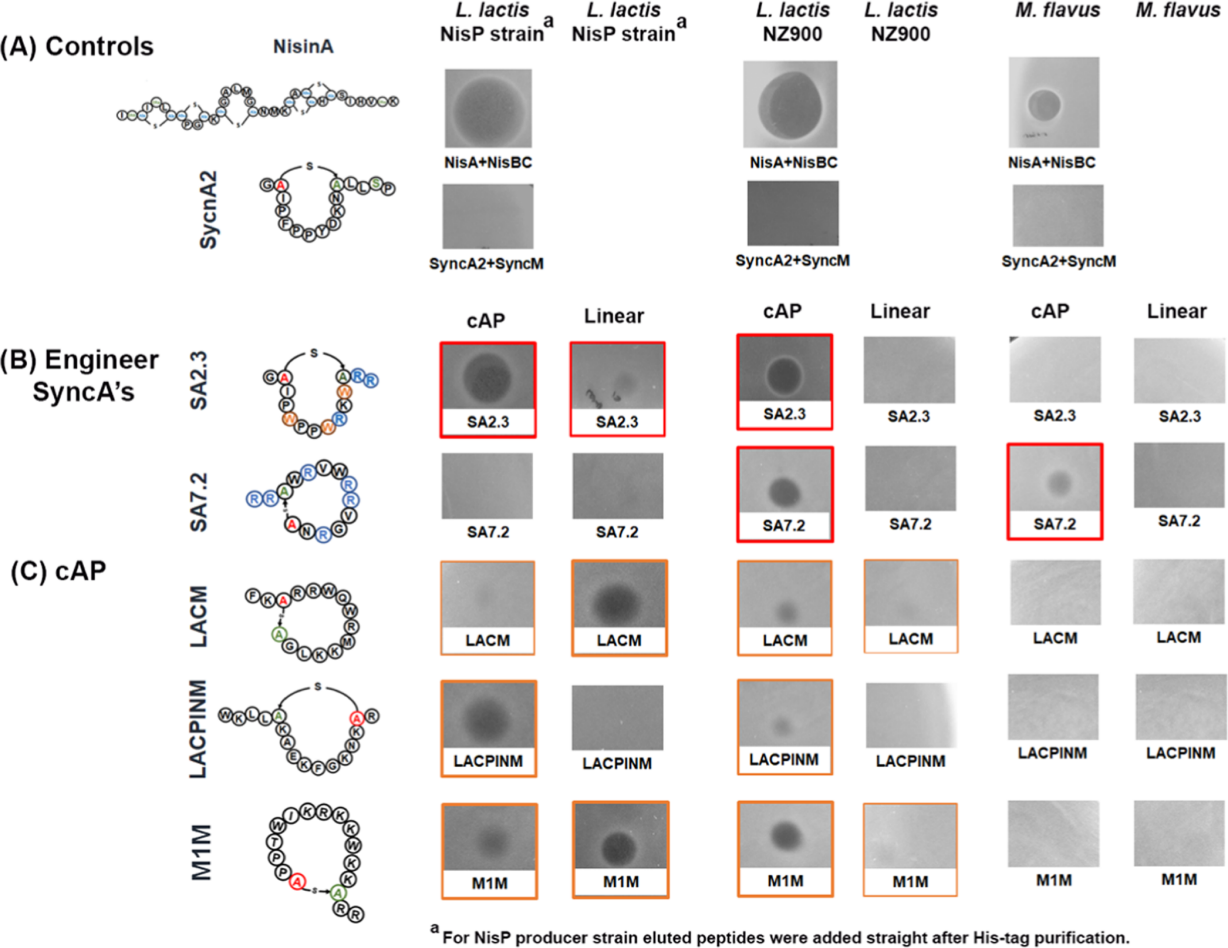
Summary of the engineered single-ringed SyncA antimicrobial activity assay and other cyclic antimicrobial peptide designs (cAP). cAP indicates the cyclized/modified peptide (peptide coexpressed with SyncM). Linear: indicates peptide expression without SyncM. We spotted 10 μL of each sample for the test. ^a^For the *L. lactis* NisP producer, His-tag elution was directly tested. Against *L. lactis* and *M. flavus*, purified cleaved core peptides were used. (A) Nisin A was spotted as a positive control and wild-type SyncA2 as a negative control. (B) Engineered SyncAs. For *L. lactis* and *M. flavus*, purified peptides were spotted with adjusted concentrations: SyncA designs: at 0.1 mg/mL except for SyncA7.2 (0.8 mg/mL). (C) Designed cyclic AP derived from l-AMP. The concentration is 0.5 mg/mL.

Once we confirmed the modification of the newly SyncA-based cAP mimics, we tested the antimicrobial activity (Figure 6) against the *L. lactis* NZ9000 NisP producer, *L. lactis* NZ9000, and *Micrococcus flavus*. We used the *L. lactis* NisP producer strain to spot the Ni-NTA His-tag elution directly, as mutants can be cleaved with the secreted NisP (except for the SyncA7-derived peptide). For *L. lactis* and *M. flavus*, HPLC-purified cleaved core peptides were tested. Only designed peptides that showed activity are presented in Figure 6. A clear inhibition zone was observed against *L. lactis* NZ9000 (both for the NisP producer and the nonproducer) for SA2.3 (Figure 6B) modified by SyncM, while the expression of the unmodified form of the SA2.3 peptide only induced a slight effect on *L. lactis* NZ9000 NisP. Antimicrobial activity was also observed for the peptide SA7.2 in *L. lactis* NZ9000 and *M. flavus* at 0.8 mg/mL (optimal minimum concentration for antimicrobial activity), while no activity was observed for the unmodified peptide (SA7.2), as shown in Figure 6B.

Overall, these results demonstrate that single-ringed SyncAs are promising candidates to rationally design new-to-nature lantibiotics. Moreover, it encourages the application of the extraordinary diversity of prochlorosin and synechococsin pools for peptide engineering and macrocycle-forming SyncM.

### Ring Insertion in Non-SyncA-Related Substrates

**Figure 7:**
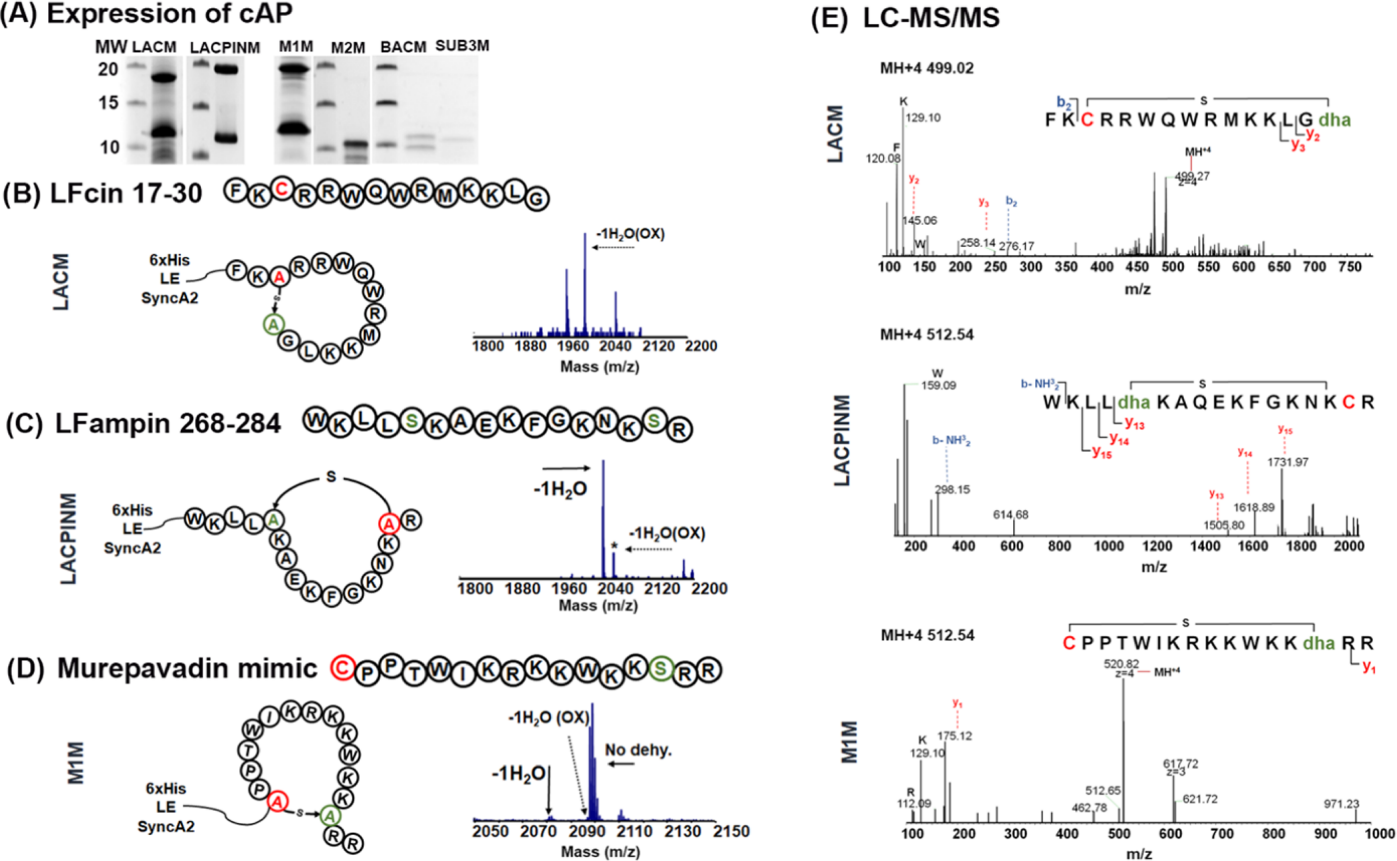
Macrocyclization of SyncM in different engineered antimicrobials. WT sequence and final expected ring installation. (A) Expression of cAP design candidates. MALDI-TOF spectra after NisP cleavage show the state of dehydration. (B) Lactoferricin-derived peptide. Wild-type name (LFcin 17–30^46^) and sequence are indicated. LACM. (C) Lactoferrampin-derived peptide. Wild-type name (LFampin 268–284^47^) and sequence are indicated. LACPINM. (D) M1M murepavadin mimic. (E) Ring insertion analysis by LC-MS/MS. LACM, LACPINM, and M1M. Insights into the dehydration and lanthionine ring pattern indicated by a black line. Dehydrated Ser/Thr is depicted in green and Cys is in red. *Mixed population with nondehydrated peptides.

Finally, we tested the ability of SyncM to insert macrocycles in substrates nonrelated to synechococsins. For this, we chose two different kinds of bioactive peptides as scaffolds, i.e., four linear antimicrobial peptides (L-AMPs) and a macrocycle antibiotic. On the one hand, AMPs are promising candidates to fight against antimicrobial resistance^42^ but their in vivo stability is limited, as they can be rapidly degraded by proteolytic enzymes.^43^ To overcome this, the introduction of macrocycles by lanthionines can confer resistance to degradation.^31^ Macrocyclic peptides produced by NRPSs are also a good source of antimicrobials.^44^ However, the complexity of their biosynthesis is often a limitation for synthesizing improved analogues. The designed cyclic antimicrobial peptides (cAPs, Table 3) were cloned behind the SyncA2 leader, coexpressed with SyncM, and purified as previously described. Of the six candidates, four were successfully produced (Figure 7A). Dehydration and cyclization occurred on three cAPs. The observed levels of dehydration and cyclization are summarized in Table 3. The level of cyclization after the NEM reaction was lower than 20%, with LACPINM being the highest and M1M being the lowest. Nondehydrated peptides and the NEM spectra of cyclized designs are shown in Figure S6A,B.

**Table 3:**
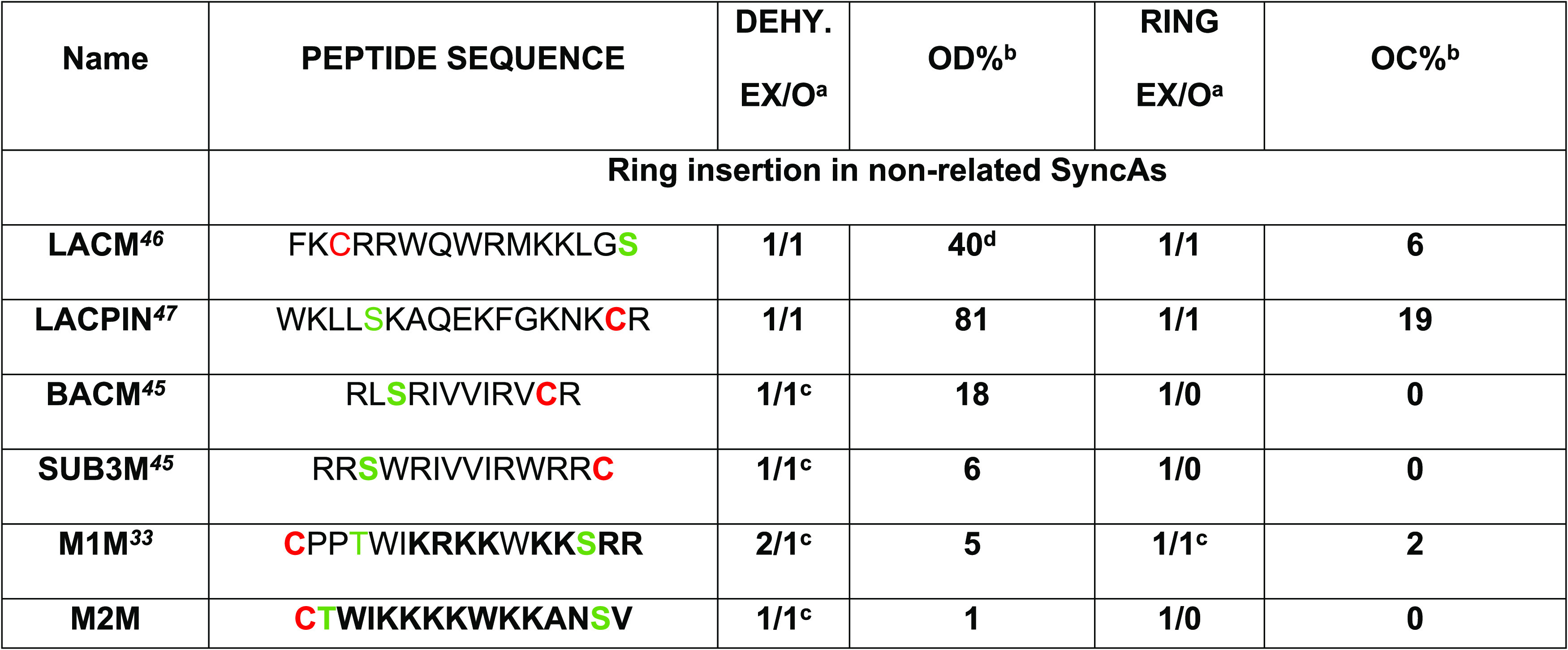
Overview of expressed His6-tag-Nonrelated SyncAs (cAP) Coexpressed with SyncM in *L. lactis* in This Work^e^

a Expected/observed dehydration state and expected/observed cyclization state.

b Percentage values were calculated from the sum of the areas of all possible forms observed in each MALDI spectra.

c Low efficiency in dehydration and/or cyclization observed.

d Intensity taken from MALDI spectra linear mode, blue line (Figure S6B—LACM).

e Ser/Thr is highlighted in green. Cys is labeled in red. Mutated residues are in bold. OD% and OC% are qualitative estimations of the observed level of dehydration and cyclization in the MALDI-TOF spectra presented in this study, respectively.

The first selected peptides were four different linear antimicrobials (Table 3): two were derived from bactenecin^45^ and two from lactoferricin^46,47^ peptides LACM (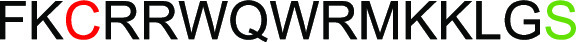) and LACPINM (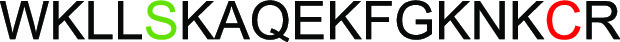; Table 1). Of the four candidates, only the lactoferricin-derived peptides were dehydrated (Figure 7B,C). Strikingly, as shown in Figure 7E, SyncM installed the expected macrocycle in these two non-SyncA-related peptides, 13- and 11-amino-acid long, respectively. For both designs, we observed a mix of oxidized and nonoxidized peptides in the dehydration analysis, which was confirmed by LC-MS (Figure S7A,B). Surprisingly, the Ser positioned at the C-terminus in LACM was successfully dehydrated, but with a cost in efficiency. It has been suggested that a Ser in this position could interfere with the processing in the case of ProcA2.8.^16^ Similarly, modification of LACM by SyncM is not efficient, due to the observation of multiple peptide intermediates (Figure S6B—LACM) from the mass spectra. Despite the possible reduction of reactivity of the C-terminal Ser, SyncM did install a ring in this substrate.

In contrast to the lactoferricin-derived peptide designs, the bactenecin-derived^45^ designs were poorly expressed and not modified by SyncM (Figure S6A) SUB3M and BACM (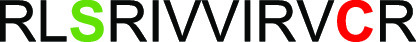). This result was unexpected, at least for SUB3M (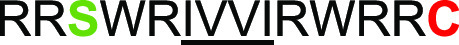), which is similar to cyclized SyncA7.2 (**RRSWR**VWRRVG**RNC**). If we compare SUB3M designs and SyncA7.2, we suspect that the −IVVI– region in SUB3M might have altered the peptide′s prefolding and affected the dehydration process, which would also explain why BACM was not modified.

The last two designs were derived from the synthetic cyclic Protegrin-1 peptidomimetic antibiotic murepavadin,^48^ used specifically against *Pseudomonas aeruginosa* (Figures 7D and S8). As murepavadin contains a single large macrocycle, two residues of the original scaffold needed to be replaced to install a lanthionine ring. We tried two designs. In the first design, M1M (CPPTWIKRKKWKKSRR), two Pro residues found in the original cyclic peptide were maintained, thereby faintly resembling the SyncA2 scaffold to ensure potential modification by SyncM. In the second design, M2M (CTWIKKKKWKKANSV), these Pro were substituted by the lanthionine-ring-forming residues to potentially structurally resemble the stable β-hairpin conformation important for the antimicrobial activity that the original d-Pro–l-Pro residues induce.^49,50^ M1M was produced at a higher yield than M2M (Figure 7A) and displayed a higher degree of dehydration than M2M. MALDI-TOF MS and LC-MS (Figures 7 and S7C) also showed a low degree of dehydration. However, fragmentation data support (Figure 7E—M1M) the presence of a macrocycle, despite the low dehydration efficiency. No ring was installed for M2M (Figure S6B). Thus, we suggest that the prolines can confer a prestructure that promotes processing by SyncM.

An antimicrobial activity test was done with the non-SyncA-related substrates against the same sensitive strains, i.e., *L. lactis* NisP producer, *L. lactis* NZ9000, and *M. flavus* (Figure 6). We spotted 10 μL of linear AMP (expressed without SyncM) and cAP (expressed with SyncM) at a final concentration of 0.5 mg/mL. The three cAPs have an ASPR motif for NisP cleavage. In the *L. lactis* NisP producer strain (Figure 6C), we observed an inhibition area for cyclic LACPIN and M1M peptides. Also, the linear form of M1M showed activity against this strain but not against *L. lactis* NZ9000, for which only cyclized M1M was active, although the weak activity of cyclized LACM and LACPINM was also observed (Figure 6C). No activity was found in *M. flavus*. Surprisingly, no evident activity was observed from the linear version of the antimicrobial lactoferrin-derived peptides against *L. lactis*. The reason for this is unclear but possibly due to peptide instability. Despite the nonefficient modification of these antimicrobial peptides, together with the results obtained from the design of SyncA-derived antimicrobial peptides, we suggest that the introduction of a lanthionine macrocycle by SyncM could indeed confer stability to the peptides.

The findings in this study show that SyncM has a relaxed specificity toward different core substrates. However, this flexibility has limitations. Moreover, we suggest that these limitations will depend on the chosen peptide. Our results support the scenario where the substrate characteristics play an essential role in the processing of the peptide.^1,27,33,51^ Therefore, the structure of the core sequence can help prevent dehydration, cyclization, or both.^33,41,51^ However, it is also possible that the interaction between SyncM and nonsuccessful designs could be due to unknown steric hindrances.^52^ Moreover, we do not discard the possibility that the chosen leader expressed with the different hybrids could also affect the result.

Taken together, these results demonstrate the ability of SyncM to form macrocycles in non-lanthipeptide molecules, with varying degrees of modification efficiency. Interestingly, we also observed clear antibacterial activity in the nonrelated SyncA antimicrobial peptide murepavadin mimic M1M. The findings presented here are a positive proof of concept of the potential of this expression system to generate novel macrocycles containing antimicrobial peptides by modification using SyncM.

## Conclusions

This paper demonstrates the applicability of SyncM for the rational design of new-to-nature lanthipeptides, particularly with a macrocyclic structure. We provide insights into the requirements for the dehydration and cyclization processes catalyzed by SyncM. We show that SyncM tolerates a large number of amino acid substitutions either inside the ring or in residues flanking Ser, Thr, and Cys. Notably, the introduction of cationic amino acids is well accepted by SyncM. Prolines can give a favorable preconformation of the peptide and could be essential for processing. However, results obtained from SyncA6 indicate that not all SyncAs will be suitable for engineering. Overall, the data presented here support the hypothesis that the core sequence confers a preorganization that contributes to the effective processing by SyncM. It can either enhance or prevent dehydration, cyclization, or both. Even though SyncM can install rings in both orientations, different substrates might have a preferential order of the Ser/Thr and Cys residues within the peptide. Cyclization with a C-terminal Ser is possible with SyncM, a rare event in the lanthipeptide family. Nevertheless, the modification efficiency is low.

Further structural studies could help better understand the interactions between SyncA substrates and SyncM that promote modification Also, it is still a challenge to identify which specific preconformation is beneficial for efficient processing. Despite the limitations found in some peptides, the findings suggest that these are case-specific and will depend on the chosen scaffold. Therefore, general modification rules for different substrates by SyncM cannot be discerned. Our study highlights the promiscuity and remarkable singularity of SyncM to install exceptionally large rings, not only in native precursors and derivatives but also in non-lanthipeptide peptides. Moreover, we show for the first time the successful design and production of a novel, fully modified, and processed SyncA-based peptide with antimicrobial activity, containing a macrocycle installed by SyncM. This result highlights the potential of these substrates for creating new-to-nature lantibiotics. However, it also requires trial-and-error experiments with different precursors to find optimal candidates. Our study broadens the scope of this RiPP-based system as a bioengineering tool for the introduction of exceptionally large macrocycles in lanthipeptides or other bioactive macrocyclic molecules, thereby paving the way for using RiPP-based modification systems toward the production of novel peptides for therapeutic applications.

## Materials and Methods

### Bacterial Strains, Plasmids, and Growth Conditions

*L. lactis* NZ9000 was used for cloning SyncA2 and SyncA6 mutant′s peptides and the coexpression with the modification enzyme SyncM.^28^*L. lactis* was grown in M17 (Difco, Le Pont de Claix, France) + 0.5% glucose (GM17) at 30 °C without shaking for genetic manipulation, protein expression, and purification assays. Chloramphenicol and/or erythromycin (Sigma-Aldrich, Darmstadt, Germany) were added at a final concentration of 5 and 10 μg/mL, respectively. For activity tests, *M. flavus* was grown in LB broth (Sigma) and incubated statically at 30 °C. A detailed list of strains and plasmids is shown in Table S2.

### Molecular Cloning

The plasmids pNZ8048_SyncA2, pNZ8048_-SyncA6, and pNZ8048_-SyncA7^28^ (Table S2) were used as templates for generating the desired mutants via site-directed mutagenesis^53,54^ protocols. Primers were ordered with phosphorylation in the 5′. Furthermore, the PCR reaction was carried out using Phusion High-Fidelity Polymerase (Thermo Fisher). We used a PCR cleaning kit (MN, Duren, Germany) to purify the PCR products. Then, a self-circularization reaction was performed with the T4 DNA ligase (Thermo Fisher, Netherlands). Ligations were dialyzed against ultrapure water and transformed in electrocompetent cells of *L. lactis* NZ9000 pTLR-SyncM^28^ using a Bio-Tad Gene Pulser (Bio-Rad, Richmond, CA). *L. lactis* NZ9000 electrocompetent cells and transformation were performed following Holo and Nes.^55^ Finally, all plasmid constructs were confirmed by DNA sequencing (Macrogen Europe, Amsterdam, Netherlands). For hybrid peptides, we selected the SyncA2-leader-ASPR. The desired sequence was cloned behind the ASPR site.

### Medium-Scale Expression and Purification of Mutants

For medium-scale expression, 400 mL of GM17 was inoculated at 1:50 dilution with overnight culture growth at 30 °C for each mutant. Cells were grown statically at 30 °C until OD_600_ reached 0.4 and induced with 5 ng/mL of nisin. After induction, the cells were grown overnight at 30 °C. The following day, the cultures were harvested by centrifugation (4 °C, 8000 rpm, 40 min), resuspended in binding buffer (20 mM NaH_2_PO_4_, 0.5 M NaCl, 30 mM imidazole, pH 7.4), and washed one time. Cells were lysed by sonication (VibraCell, 30 s ON, 10 s OFF, 75% amplitude, 15 min). Purification was performed with the Ni-NTA agarose (QIAGEN, United States) column protocol followed in Arias-Orozco et al.^28^ His-tag elutions were filtrated through a 0.2 μm filter and purified by a reverse-phase C18 (Phenomenex Aeris 250 × 4.6 mm^2^, 3.6 μm particle size, 100 Å pore size) in an Agilent Infinity HPLC system. HPCL was first run at 5% solvent B (100% acetonitrile: 0.1% trifluoroacetic acid) and 95% solvent A for 10 min, then a linear gradient from 20 to 60% solvent B, and a final step of 100% solvent B. SyncA6 and SA7.2 were purified with the same program but using a C4 column. Finally, fractions were collected, lyophilized, weighed, and resuspended in solvent A (ultrapure water plus 0.1% TFA) and analyzed by matrix-assisted laser desorption/ionization with a time-of-flight detector (MALDI-TOF). For the MALDI-TOF, each sample (1 μL) was spotted on the target and dried at room temperature. Then, 0.5 μL of the α-cyano-4-hydroxycinnamic acid matrix (3 mg/mL) was spotted on the samples. Once the samples were dried, mass spectrometry analysis was performed using a 4800 plus MALDI/TOF analyzer (Applied Biosystems) operated in MS linear mid-mass positive mode. The MS reflector mode was applied for peptides presented in the main study.

For the leader release, the fractions with pure full peptides were freeze-dried and resuspended in 1 mL of 100 mM Tris-HCL, pH 6. Then, purified NisP was added as indicated by Montalbán-López et al.^56^ (1:10 ratio) and incubated at 30 °C for 2 h.^52^ For SyncA7 variants, the sample was resuspended in 50 mM Tris pH 8 and 1 mM DTT. Then, the cleavage reaction mix containing (as recommended by the authors^38^) ∼10 μM LaTh150 protease and ∼100 μM substrate was incubated overnight at room temperature.

### MS Dehydration and Cyclization Analysis

The dehydration/cyclization levels of the core peptides were analyzed by MALDI-TOF. The cyclization reaction was performed following the provider protocol. NEM (N-ethylmaleimide) was dissolved in water (150 mM) and added to a reaction mix with 1× PBS (pH 7.2) with a minimum of 10-fold molar excess plus 5 μL of the peptide with a maximum final concentration of ∼150 μM.

### LC-MS/MS

The protocol was followed as in our previous study.^28^ The equipment used was an Ultimate 3000 nano-HPLC system (Thermo Fisher Scientific) coupled online to a Q-Exactive-Plus mass spectrometer with a NanoFlex source (Thermo Fisher Scientific) equipped with a stainless steel emitter. A C18 column PepMAP100 2 mm particles (Dionex) was used. A mobile-phase gradient was followed at a flow rate of 300 nL/min: 2–85% solvent B in 60 min, 85% B for 5 min, back to 2% B in 1 min, and held at 2% B for 15 min. Solvent A was 100:0 H_2_O/acetonitrile (v/v) with 0.1% formic acid, and solvent B was 0:100 H_2_O/acetonitrile (v/v) with 0.1% formic acid.

MS data were acquired using a data-dependent top-10 method, dynamically choosing the most abundant not-yet-sequenced precursor ions from the survey scans (300–2000 Th) with a dynamic exclusion of 5 s. Sequencing was performed via higher-energy collisional dissociation fragmentation with a target value of 1e4 ions determined with predictive automatic gain control. Isolation of precursors was performed with a window of 2 Da. Survey scans were acquired at a resolution of 70 000 at *m*/*z* 200. Resolution for HCD spectra was set to 17 500 at *m*/*z* 200 with a maximum ion injection time of 100 ms. The normalized collision energy was set at 28. Furthermore, the S-lens RF level was set at 60, and the capillary temperature was set at 250 °C. Precursor ions with single, unassigned, or five and higher charge states were excluded from fragmentation selection.

### Antimicrobial Activity Assays in Agar

To test the antimicrobial activity in solid media, we prepared 0.75% (w/v) agar of LB (*M. flavus*) and GM17 (*L. lactis* strains) and cooled it to ∼45 °C. After that, we added 300 μL of overnight inoculum to 30 mL of medium and mixed it. The mix was poured onto the plates and dried for ∼30 min at the flame. Once dried, we spotted 10 μL of each peptide. When the spot was dried, the plates were transferred to 30 °C and incubated overnight.

## Supporting Information Available

SA2-SWAP extra data; LC-MS/MS macrocycles; LC-MS of mutants; ring analysis with the NEM MALDI-TOF spectrum of specific mutants; murepavadin and mimic chemical structure; table of core peptide amino acid profile; and table summary of plasmids and strains used in this study (PDF)
