# Supplementary material for: Investigating the Specificity of the Dehydration and Cyclization Reactions in Engineered Lanthipeptides by Synechococcal SyncM
Source: ACS Synth Biol. 2022 Dec 15;12(1):164–77. doi: 10.1021/acssynbio.2c00455 (PMC9872173; doi:10.1021/acssynbio.2c00455)
Supplement: Supplementary file 1 — sb2c00455_si_001.pdf [file sb2c00455_si_001.pdf]

# Investigating the specificity of the dehydration and cyclization reactions in engineered lanthipeptides by synechococcal SyncM

Patricia Arias-Orozco[1], Yunhai Yi[1], Fleur Ruijne, Rubén Cebrián [1,2] and Oscar P. Kuipers[1]*

[1]Department of Molecular Genetics, University of Groningen, Groningen, Nijenborgh 7, 9747AG, Groningen, The Netherlands.
[2]Department of Clinical Microbiology, Instituto de Investigación Biosanitaria ibs. GRANADA, University Hospital Clínico San Cecilio, Av. De la Innovación s/n, 18016, Granada, Spain.
*Corresponding author: Oscar P. Kuipers, o.p.kuipers@rug.nl

**Supporting Information for Publication**

**FIGURES AND TABLES**

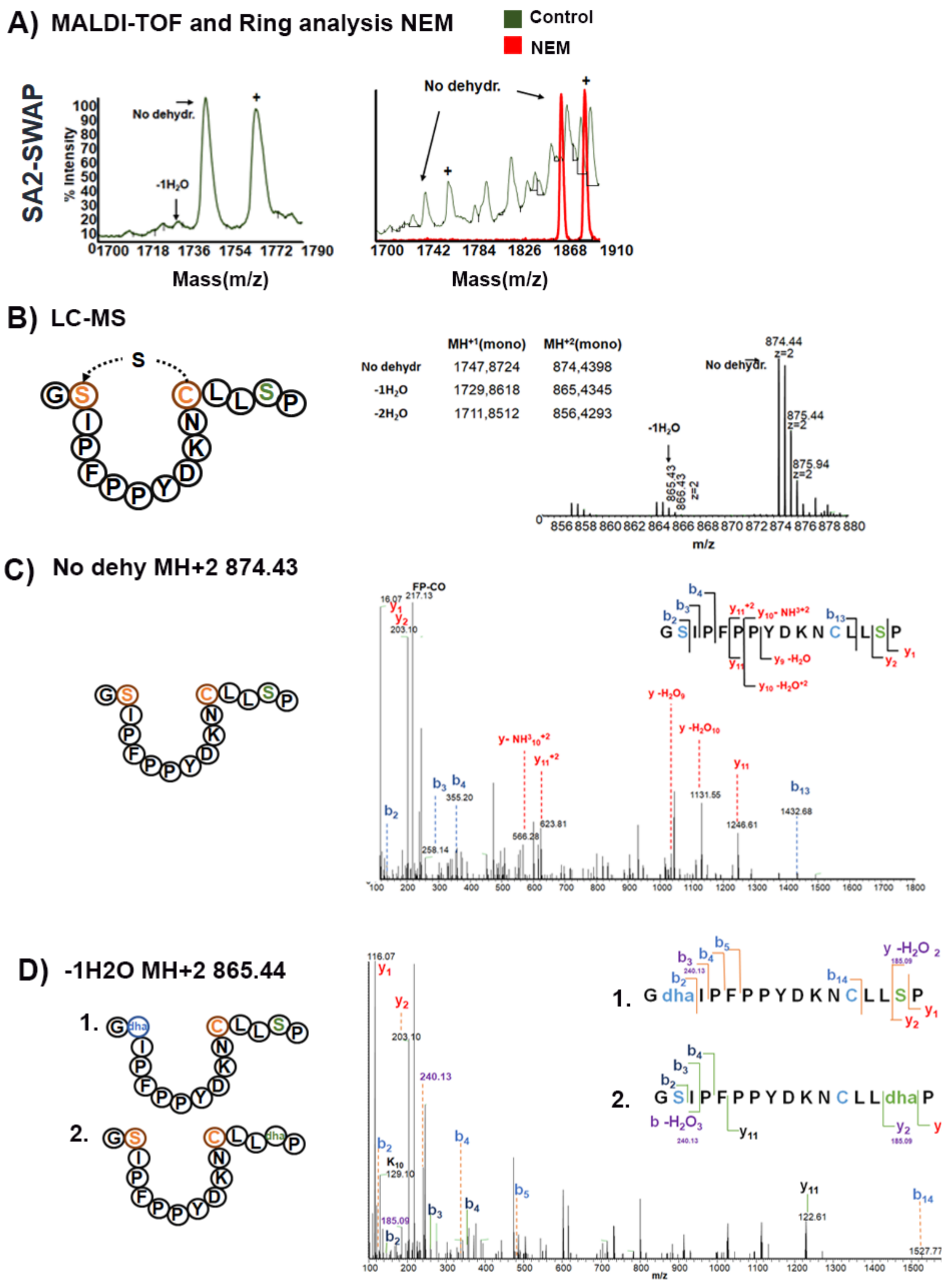


**Supplementary Figure S1**. Overview of SA2-SWAP of SyncA2 mutants. Dehydration and cyclization state analysis. A) MALDI-TOF spectra of SA2-SWAP; the dehydration state is depicted by arrows. Ring analysis with N-ethylmaleimide (NEM, red spectra correspond after NEM reaction). (+) Na-adduct B) LC-MS spectra. Detected masses

were found to correspond to non-dehydrated and -1$H_2O$ peptides. C) $MS^2$ spectra of the non-dehydrated peptide. Ion fragments of the unmodified peptide are indicated. D) $MS^2$ spectra of the -1$H_2O$ peptide. Ion fragments of -1$H_2O$ unmodified peptide are indicated. Two options for dehydration were found. Form 1 with the S2 dehydrated. Form 2 with the S15 dehydrated. Ion fragments of each form are differentiated with color. (1-Orange, 2-Dark blue, same ion-Red). Masses that could correspond to both peptides are identified in purple.

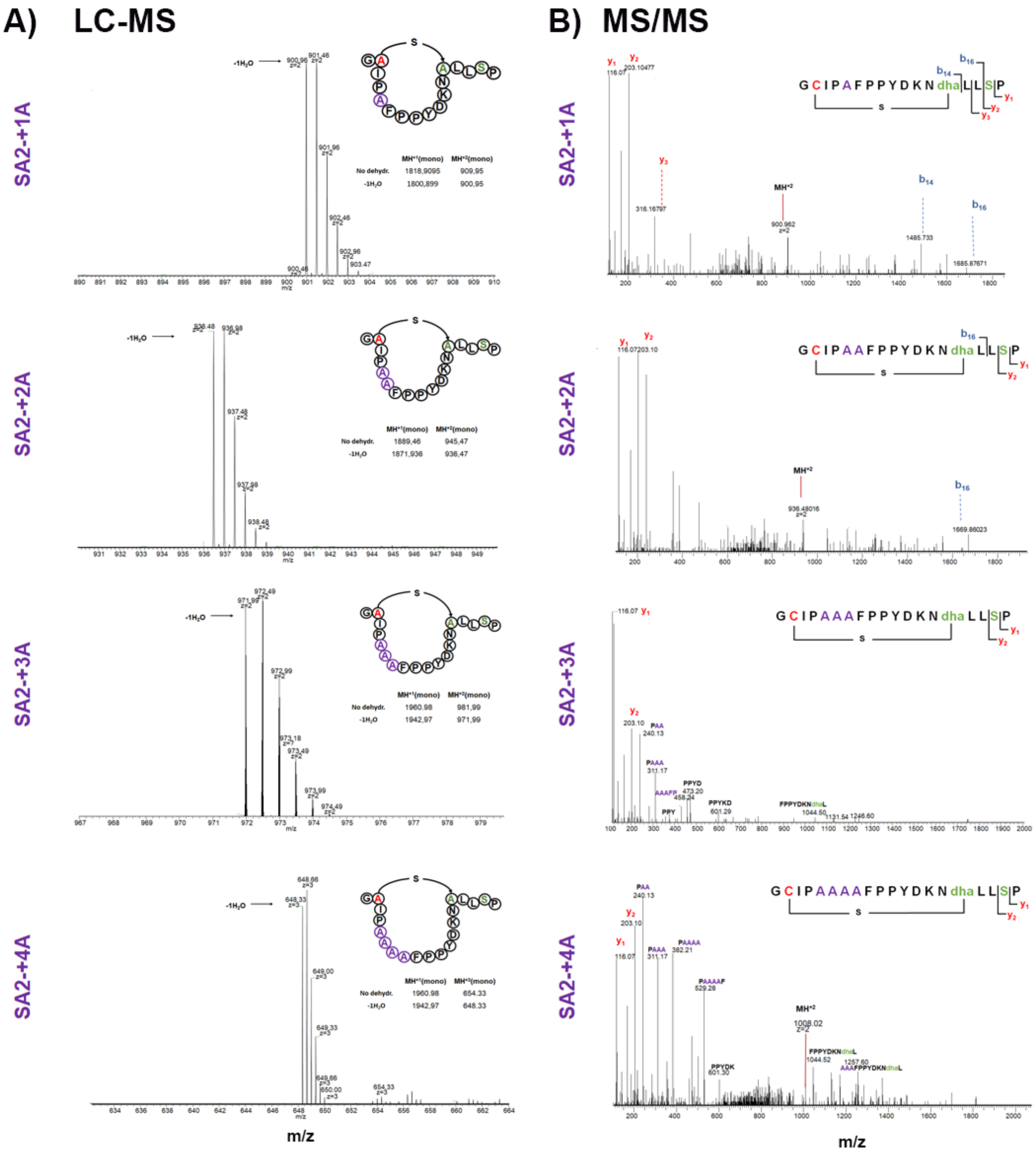


**Supplementary Figure S2**. LC-MS/MS spectra of SyncA2 macrocycle variants. A) Shows the LC-MS chromatograms. Expected dehydration was found in all mutants. SA2-+1A, SA2-+2A, SA2-+3A and SA2-+4[a]. B) Ring formation analysis showing MS$^2$ spectra of each macrocycle mutant: SA2-+1A, SA2-+2A, SA2-+3A, and SA2-+4[a] Fragmented ions, corresponding with the two-time mass charge in each mutant, are depicted. A lanthionine ring was installed by SyncM in all variants.

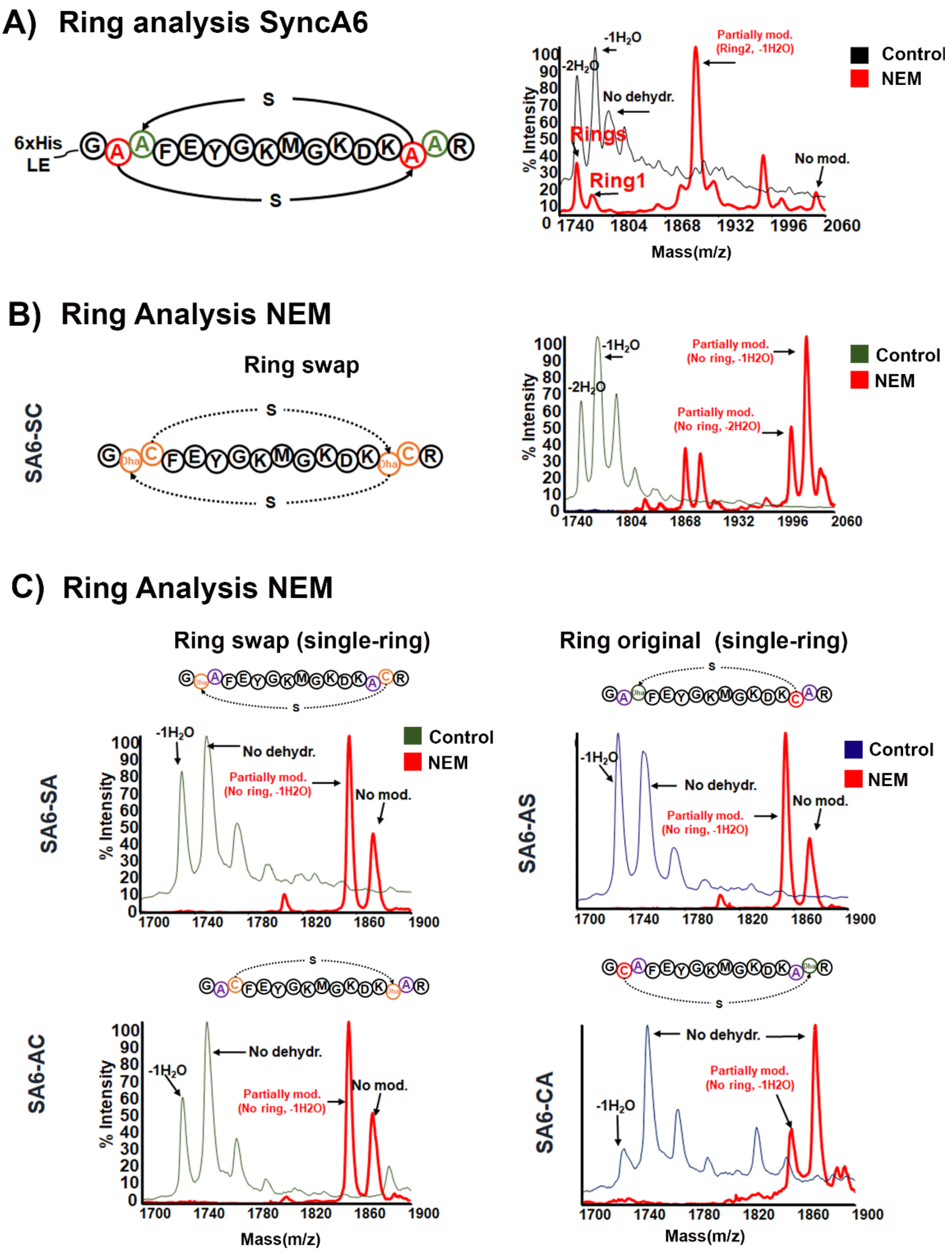


**Supplementary Figure S3.** Ring analysis of SyncA6 mutants with the NEM reaction. A) SyncA6 control. B) Mutants with a change in the dehydration and ring direction. C) Single-ring mutants. Red lines correspond to spectra after NEM reaction and back line

(black, green and blue) depicts control spectra. No ring was formed in any of the mutants.

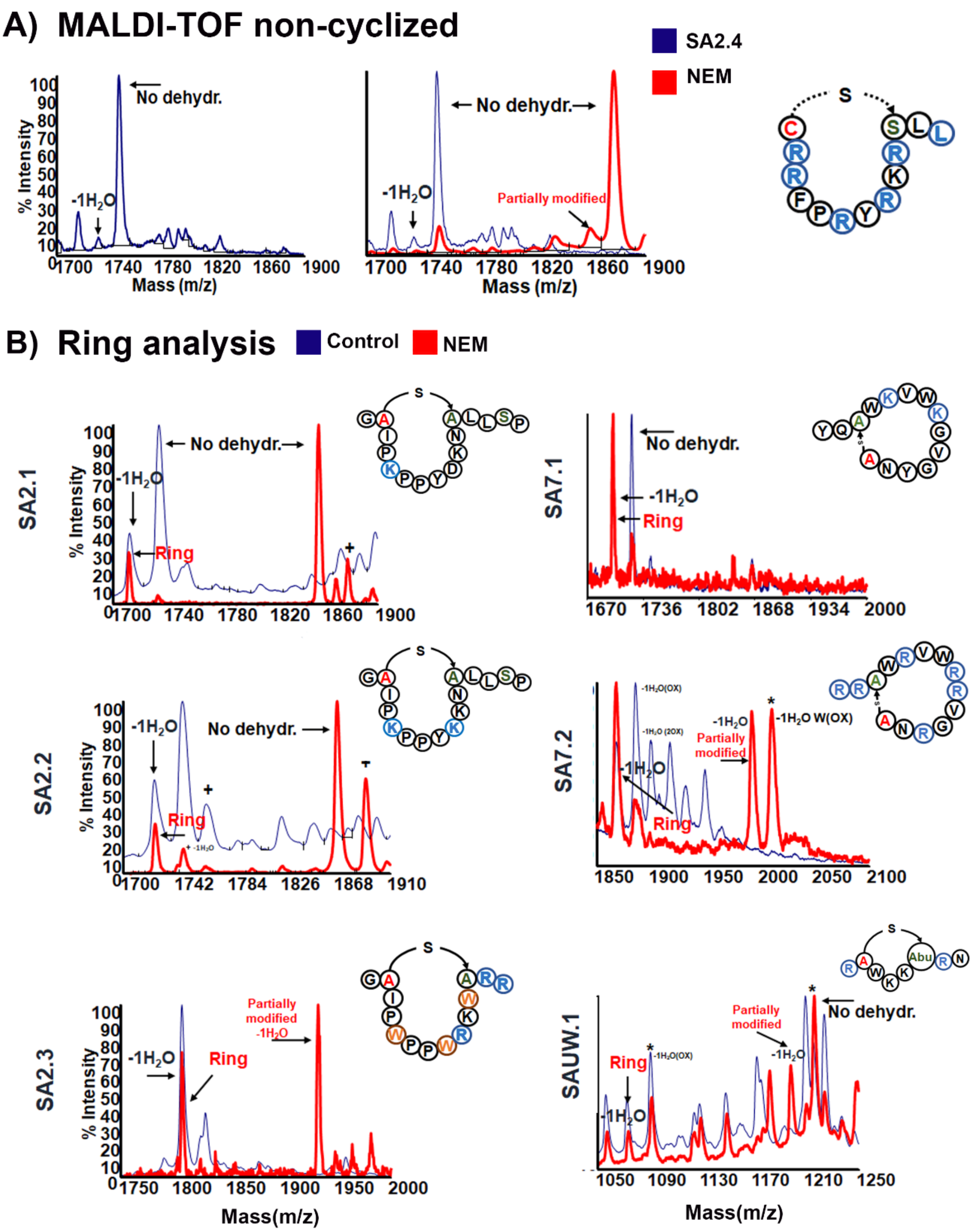


**Supplementary Figure S4**. Ring analysis of single-ringed engineered SyncA's with the NEM reaction. (+) Na adduct . A) The SA2.4 MALDI-TOF dehydration state is

depicted by arrows. Ring analysis with N-ethylmaleimide (NEM, red spectra correspond to the situation after NEM reaction). B) Single-ring variants with a ring installed. SA2.1, SA2.2, SA2.3, SA7.1, SA7.2, SAUW.1.

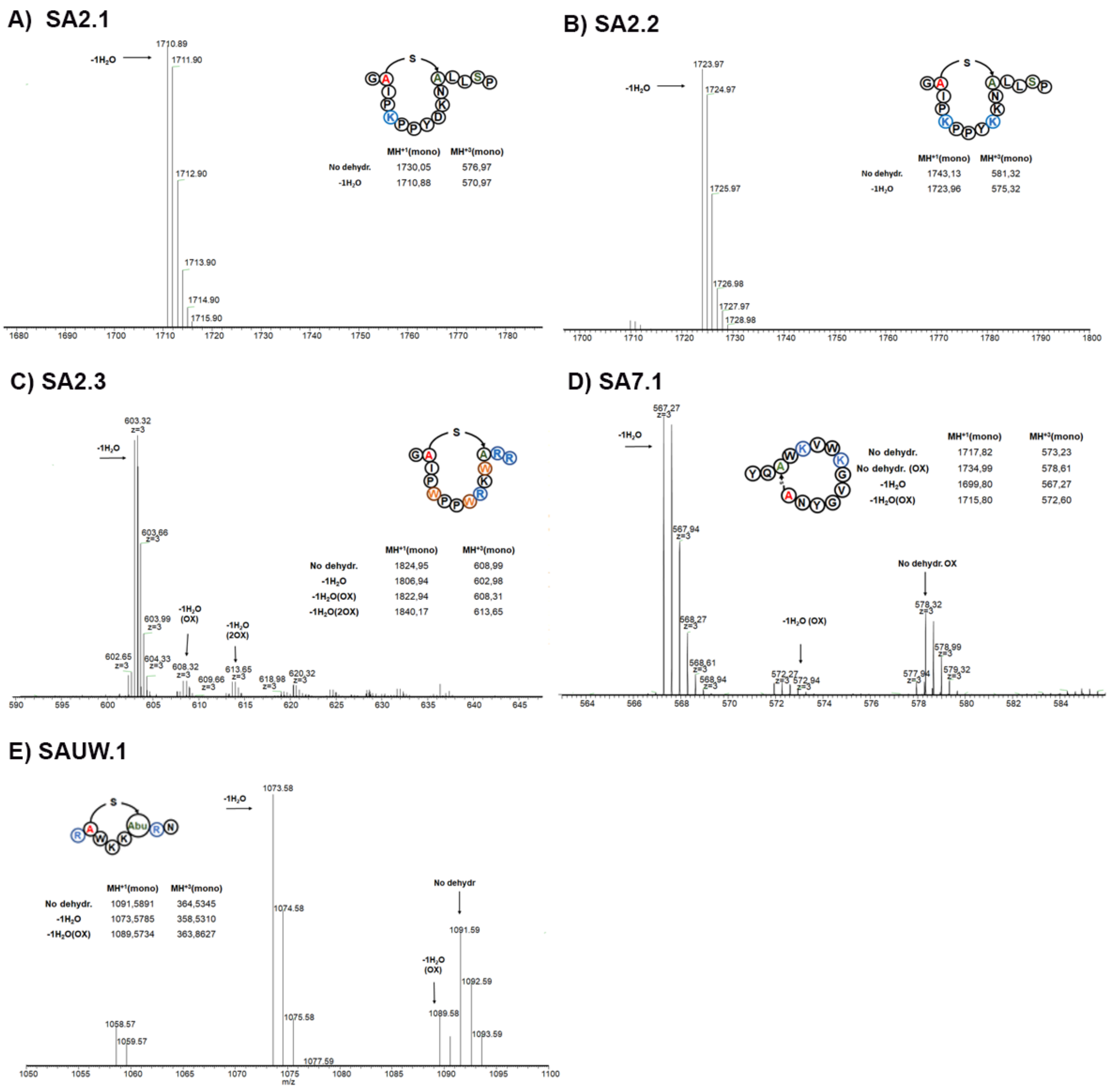


**Supplementary Figure S5.** LC-MS Spectra of SyncA2 engineered single-ringed SyncA's. A) SA2.1 B) SA2.2, C) SA2.3, D) SA7. E) SAUW.1.

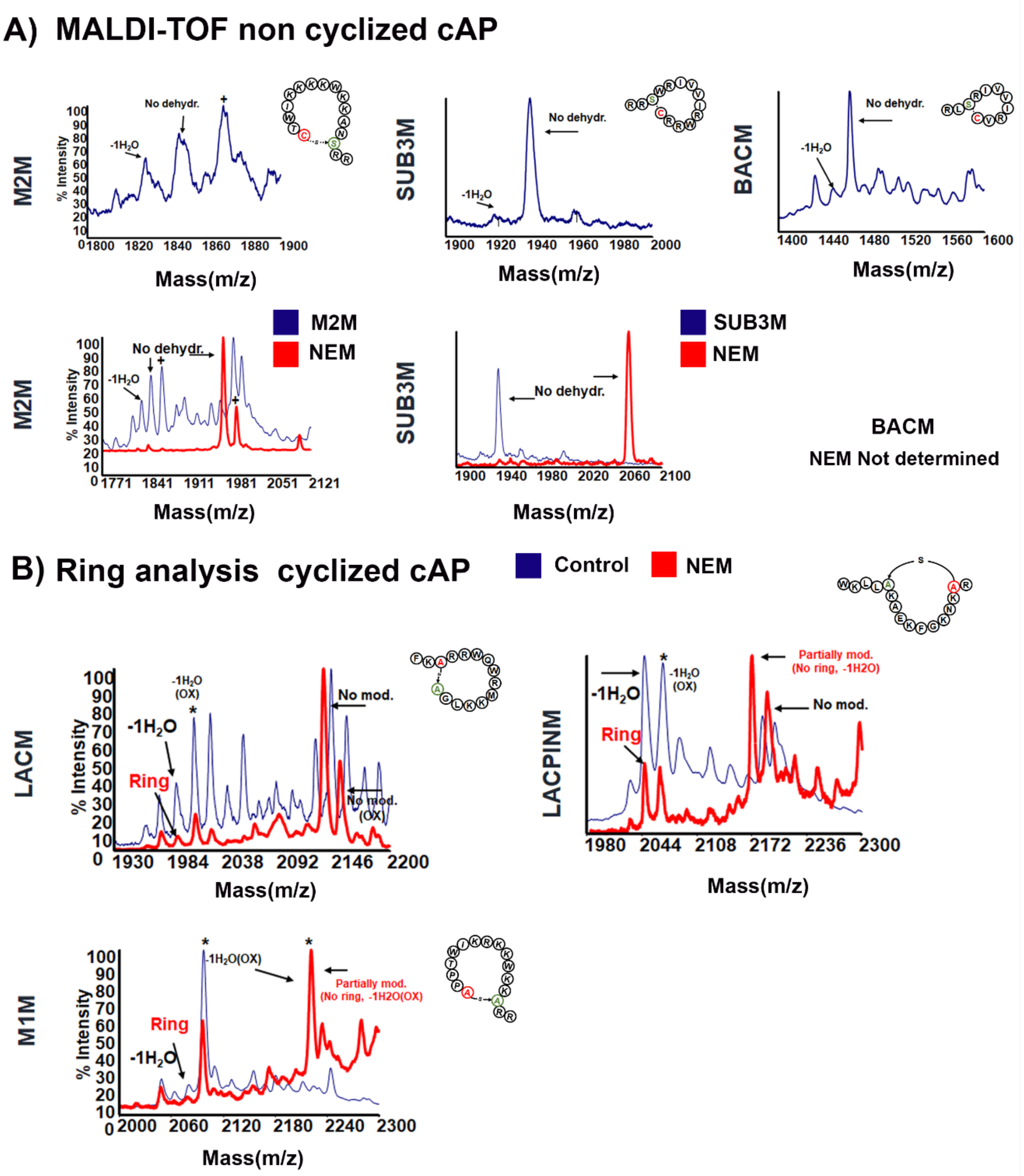


**Supplementary Figure S6**. Ring analysis of non-prochlorosin peptides co-expressed with SyncM. A) MALDI-TOF and NEM of non-modified AMPs B) Ring analysis of cAMP with NEM. LACM, M1M, and LACPINM.

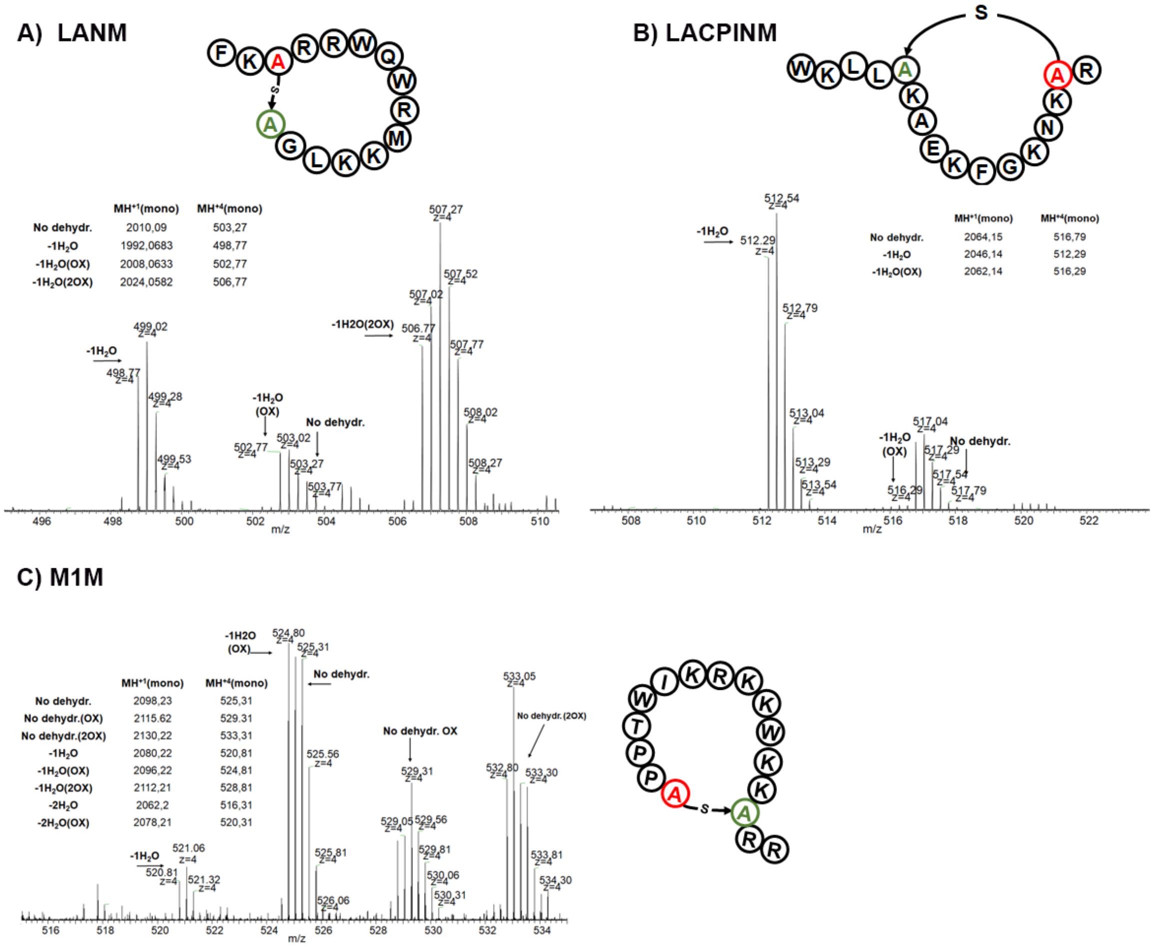


**Supplementary Figure S7** LC-MS analysis of non-prochlorosin peptides co-expressed with SyncM A) LACTO_+S15, B) LACPIN_S16C- and C) SyncAUW.1.

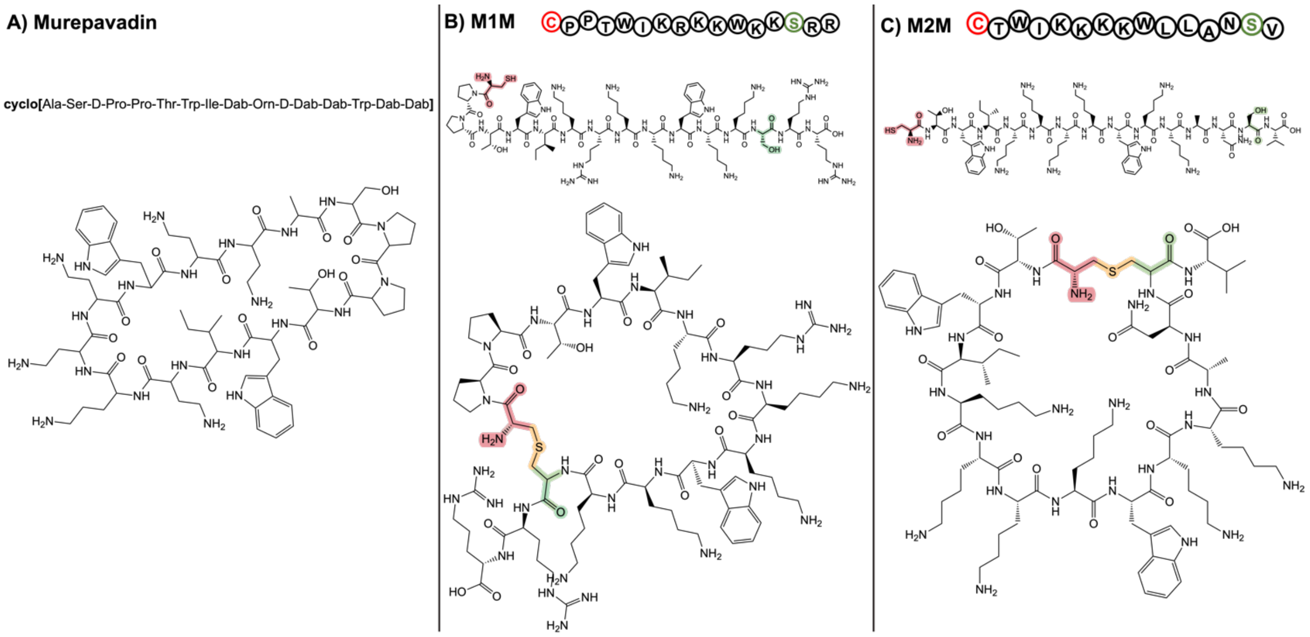


**Supplementary figure S8. Chemical structure of Murepavadin[1] and proposed chemical structure of M1M and M2M with the lanthionine ring.** A) Murepavadin sequence and known structure; B) M1M amino acid sequence, linear chemical structure Cys (Highlighted in Red) and Ser (Highlighted in green) and macrocyclic structure after SyncM (lanthionine ring in yellow); C) M1M amino acid sequence, linear chemical structure Cys (Highlighted in Red) and Ser (Highlighted in green) and macrocyclic structure after SyncM (lanthionine ring in yellow).

**Supplementary Table S1**. Core peptide sequences and amino acid profiles of 79 SyncA's from *Synechococcus* MI9509. Residues involved in ring formation are highlighted in selected peptides (red for (C) and green for (T/S)). Blue numbers highest occurrence and red numbers lowest occurrence.

| # | Core Peptide Sequence | AA[a] | Ala | Ile | Val | Leu | Met | Phe | Trp | Tyr | Pro | Gly | Cys | Ser | Thr | Asn | Gln | Arg | Lys | His | Asp | Glu | CHARGE |
|---|---|---|---|---|---|---|---|---|---|---|---|---|---|---|---|---|---|---|---|---|---|---|---|
| **1** | TIPPIVNWIGEKMDKGCAGTLFAASYTSCVAMGTANKECG | 40 | 5 | 3 | 2 | 1 | 2 | 1 | 1 | 1 | 2 | 5 | 3 | 2 | 4 | 2 | 0 | 0 | 3 | 0 | 1 | 2 | 0 |
| **SA1** | AGSANPQGGDPWCTWDFPICINHK | 24 | 2 | 2 | 0 | 0 | 0 | 1 | 2 | 0 | 3 | 3 | 2 | 1 | 1 | 2 | 1 | 0 | 1 | 1 | 2 | 0 | 1 |
| **SA2** | **GCIPFPPYDKNSLLSP** | 16 | 0 | 1 | 0 | 2 | 0 | 1 | 0 | 1 | 4 | 1 | 1 | 2 | 0 | 1 | 0 | 0 | 1 | 0 | 1 | 0 | 0 |
| **4** | RRCDSCGIWGWIGMAKTCKGPTQG | 24 | 1 | 2 | 0 | 0 | 1 | 0 | 2 | 0 | 1 | 5 | 3 | 1 | 2 | 0 | 1 | 2 | 2 | 0 | 1 | 0 | 3 |
| **5** | IPGVATTPQLTHVLSNLTSQNPDFFNGFDFFSSLTNPIDKPPIPGTIERINYLLKTLQDIVAITVKDSQYPYQKSTSTLSCFAIDGR | 96 | 3 | 8 | 4 | 9 | 0 | 6 | 0 | 3 | 8 | 4 | 2 | 10 | 11 | 5 | 7 | 3 | 5 | 1 | 6 | 1 | 1 |
| **6** | QQLTSRKSCGDCVLGRALSCLLLGSYAKQKR | 22 | 2 | 0 | 1 | 5 | 0 | 0 | 0 | 1 | 0 | 3 | 2 | 2 | 0 | 0 | 1 | 2 | 2 | 0 | 1 | 0 | 3 |
| **7** | **TNILNSAGFCNDNPESRSLQP** | 21 | 1 | 1 | 0 | 2 | 0 | 1 | 0 | 0 | 2 | 1 | 1 | 3 | 1 | 4 | 1 | 1 | 0 | 0 | 1 | 1 | -1 |
| **8** | EYECLCHCTWDDVTSCTVNTWGLG | 0 | 0 | 0 | 2 | 2 | 0 | 0 | 2 | 1 | 0 | 2 | 0 | 0 | 0 | 0 | 0 | 0 | 0 | 0 | 0 | 0 | -4 |

| | | | | | | | | | | | | | | | | | | | | | | | |
|---|---|---|---|---|---|---|---|---|---|---|---|---|---|---|---|---|---|---|---|---|---|---|---|
| **9** | GACPSTFTIDACAATGCNPKDL | 22 | 4 | 1 | 0 | 1 | 0 | 1 | 0 | 0 | 2 | 2 | 3 | 1 | 3 | 1 | 0 | 0 | 1 | 0 | 2 | 0 | -1 |
| **10** | AASAIVGDCQSQEGHVCFSEGEVVCGKR | 28 | 3 | 1 | 4 | 0 | 0 | 1 | 0 | 0 | 0 | 4 | 3 | 3 | 0 | 0 | 2 | 1 | 1 | 1 | 1 | 3 | -2 |
| **11** | **TAWSNSCY** | 8 | 1 | 0 | 0 | 0 | 0 | 0 | 1 | 1 | 0 | 0 | 1 | 2 | 1 | 1 | 0 | 0 | 0 | 0 | 0 | 0 | 0 |
| **12** | CDIELVSLCSKTHNVLC | 17 | 0 | 1 | 2 | 3 | 0 | 0 | 0 | 0 | 0 | 0 | 3 | 2 | 1 | 1 | 0 | 0 | 1 | 1 | 1 | 1 | -1 |
| **13** | GLSIWGCTECVDGTFHCKRCDLSNLF | 26 | 0 | 1 | 1 | 3 | 0 | 2 | 1 | 0 | 0 | 3 | 4 | 2 | 2 | 1 | 0 | 1 | 1 | 1 | 2 | 1 | -1 |
| **14** | NSWGWCRGFTGLEGCSAWCNRFCHGRQTR | 29 | 1 | 0 | 0 | 1 | 0 | 2 | 3 | 0 | 0 | 5 | 4 | 2 | 2 | 2 | 1 | 4 | 0 | 1 | 0 | 1 | 3 |
| **15** | EYTGNDTGPTNMYGVCHIPKCPEWAASKG | 29 | 2 | 1 | 1 | 0 | 1 | 0 | 1 | 2 | 3 | 4 | 2 | 1 | 3 | 2 | 0 | 0 | 2 | 1 | 1 | 2 | -1 |
| **16** | SGWAGYKPGQACLGVDTMAPPSPGEF | 26 | 3 | 0 | 1 | 1 | 1 | 1 | 1 | 1 | 4 | 5 | 1 | 2 | 1 | 0 | 1 | 0 | 1 | 0 | 1 | 1 | -1 |
| **SA9** | YLSYDGSERTFGCGGKGCGWVEPPTKIGGGECRDNKLR | 38 | 0 | 1 | 1 | 2 | 0 | 1 | 1 | 2 | 2 | 9 | 3 | 2 | 2 | 1 | 0 | 3 | 3 | 0 | 2 | 3 | 1 |
| **18** | GVRGGTAAYMCPTDRNKTCKPVATCPGLK | 21 | 3 | 0 | 2 | 1 | 1 | 0 | 0 | 1 | 3 | 4 | 1 | 1 | 0 | 0 | 0 | 0 | 0 | 0 | 0 | 0 | 4 |
| **19** | AEGGWCQLFPVPTACQVTWN | 20 | 2 | 0 | 2 | 1 | 0 | 1 | 2 | 0 | 2 | 2 | 2 | 0 | 2 | 1 | 2 | 0 | 0 | 0 | 0 | 1 | -1 |
| **20** | DSGGDYLSYAGCDTGRCGACSWGCPG | 26 | 2 | 0 | 0 | 1 | 0 | 0 | 1 | 2 | 1 | 7 | 4 | 3 | 1 | 0 | 0 | 1 | 0 | 0 | 3 | 0 | -2 |
| **21** | LDALKQCNFTGTCWDTLL | 18 | 1 | 0 | 0 | 4 | 0 | 1 | 1 | 0 | 0 | 1 | 2 | 0 | 3 | 1 | 1 | 0 | 1 | 0 | 2 | 0 | -1 |
| **SA18** | **SDTNWITEVSKCPWWRRS** | 18 | 0 | 1 | 1 | 0 | 0 | 0 | 3 | 0 | 1 | 0 | 1 | 3 | 2 | 1 | 0 | 2 | 1 | 0 | 1 | 1 | 1 |
| **SA13** | CWMNTCWGYGPGTAAAPDWN | 20 | 3 | 0 | 0 | 0 | 1 | 0 | 3 | 1 | 2 | 3 | 2 | 0 | 2 | 2 | 0 | 0 | 0 | 0 | 1 | 0 | -1 |

| | | | | | | | | | | | | | | | | | | | | | | | |
|---|---|---|---|---|---|---|---|---|---|---|---|---|---|---|---|---|---|---|---|---|---|---|---|
| **SA14** | TDNALNKCT<br>TGCSPSGEV<br>PGYTKPCDN<br>AII | 30 | 2 | 2 | 1 | 1 | 0 | 0 | 0 | 1 | 3 | 3 | 3 | 2 | 4 | 3 | 0 | 0 | 2 | 0 | 2 | 1 | -1 |
| **25** | ANICTGCQIA<br>TTCIDDSQIL<br>QYTNSG | 26 | 2 | 4 | 0 | 1 | 0 | 0 | 0 | 1 | 0 | 2 | 3 | 2 | 4 | 2 | 3 | 0 | 0 | 0 | 2 | 0 | -2 |
| **26** | GACEVLTGP<br>VIESYVGDS<br>WHDYCCRIT | 27 | 1 | 2 | 3 | 1 | 0 | 0 | 1 | 2 | 1 | 3 | 3 | 2 | 2 | 0 | 0 | 1 | 0 | 1 | 2 | 2 | -3 |
| **SA4** | EGSWRAGW<br>GPCRVRLSM<br>SPQHKNCRG | 26 | 1 | 0 | 1 | 1 | 1 | 0 | 2 | 0 | 2 | 4 | 2 | 3 | 0 | 1 | 1 | 4 | 1 | 1 | 0 | 1 | 4 |
| **SA5** | EQCPLNTGC<br>PLHTGWWC<br>TGVLVDQQP<br>HRSAASNI | 40 | 2 | 1 | 2 | 3 | 0 | 0 | 2 | 0 | 3 | 3 | 3 | 2 | 4 | 2 | 0 | 0 | 3 | 0 | 1 | 2 | -1 |
| **SA8** | **SDDETLTDF<br>GCKDYERY** | 17 | 0 | 0 | 0 | 1 | 0 | 1 | 0 | 2 | 0 | 1 | 1 | 1 | 2 | 0 | 0 | 1 | 1 | 0 | 4 | 2 | -4 |
| **SA3** | **NKSHYYCEP<br>IPAPFA** | 15 | 2 | 1 | 0 | 0 | 0 | 1 | 0 | 2 | 3 | 0 | 1 | 1 | 0 | 1 | 0 | 0 | 1 | 1 | 0 | 1 | 0 |
| **31** | EPTPWDPE<br>WYN | 11 | 0 | 0 | 0 | 0 | 0 | 0 | 2 | 1 | 3 | 0 | 0 | 0 | 1 | 1 | 0 | 0 | 0 | 0 | 1 | 2 | -3 |
| **32** | ECGNVSCVN<br>IYLLTLGKDK<br>EVEL | 23 | 0 | 1 | 3 | 4 | 0 | 0 | 0 | 1 | 0 | 2 | 2 | 1 | 1 | 2 | 0 | 0 | 2 | 0 | 1 | 3 | -2 |
| **33** | KVSDFCTSDI<br>VSYCCW | 16 | 0 | 1 | 2 | 0 | 0 | 1 | 1 | 1 | 0 | 0 | 3 | 3 | 1 | 0 | 0 | 0 | 1 | 0 | 2 | 0 | -1 |
| **34** | ASWSGPMW<br>NTTCIGG | 15 | 1 | 1 | 0 | 0 | 1 | 0 | 2 | 0 | 1 | 3 | 1 | 2 | 2 | 1 | 0 | 0 | 0 | 0 | 0 | 0 | 0 |
| **35** | DYCTVETAT<br>VCYIREPGC<br>DTSDYGC | 25 | 1 | 1 | 2 | 0 | 0 | 0 | 0 | 3 | 1 | 2 | 4 | 1 | 4 | 0 | 0 | 1 | 0 | 0 | 3 | 2 | -4 |
| **36** | RSYNWASW<br>CSE | 11 | 1 | 0 | 0 | 0 | 0 | 0 | 2 | 1 | 0 | 0 | 1 | 3 | 0 | 1 | 0 | 1 | 0 | 0 | 0 | 1 | 0 |
| **SA12** | YYCACTRIPV<br>YNHTN | 15 | 1 | 1 | 1 | 0 | 0 | 0 | 0 | 3 | 1 | 0 | 2 | 0 | 2 | 2 | 0 | 1 | 0 | 1 | 0 | 0 | 1 |
| **38** | GTGRHTCGT<br>GSCGCFCSI<br>YEC | 24 | 0 | 1 | 0 | 0 | 0 | 1 | 0 | 1 | 0 | 5 | 2 | 1 | 1 | 2 | 1 | 0 | 1 | 1 | 2 | 0 | 0 |
| **39** | ICRQSCIWRS<br>IFKAAPE | 17 | 2 | 3 | 0 | 0 | 0 | 1 | 1 | 0 | 1 | 0 | 2 | 2 | 0 | 0 | 1 | 2 | 1 | 0 | 0 | 1 | 2 |
| **4** | VGGVCGAFT<br>SEANGTP | 16 | 2 | 0 | 2 | 0 | 0 | 1 | 0 | 0 | 1 | 4 | 1 | 1 | 2 | 1 | 0 | 0 | 0 | 0 | 0 | 1 | -1 |
| **41** | GTDVNHCYF<br>ATAYGPHTC<br>SGACAK | 24 | 4 | 0 | 1 | 0 | 0 | 1 | 0 | 2 | 1 | 3 | 3 | 1 | 3 | 1 | 0 | 0 | 1 | 2 | 1 | 0 | 0 |
| **42** | TACALRVGT<br>VFIGSCWN | 17 | 2 | 1 | 2 | 1 | 0 | 1 | 1 | 0 | 0 | 2 | 2 | 1 | 2 | 1 | 0 | 1 | 0 | 0 | 0 | 0 | 1 |

| | | | | | | | | | | | | | | | | | | | | | | | |
|---|---|---|---|---|---|---|---|---|---|---|---|---|---|---|---|---|---|---|---|---|---|---|---|
| **43** | DLHSVCGFSACGTCGLEGTCLICEDT | 26 | 1 | 1 | 1 | 3 | 0 | 1 | 0 | 0 | 0 | 4 | 5 | 2 | 3 | 0 | 0 | 0 | 0 | 1 | 2 | 2 | -4 |
| **44** | LAWGAGGSRVPIN | 13 | 2 | 1 | 1 | 1 | 0 | 0 | 1 | 0 | 1 | 3 | 0 | 1 | 0 | 1 | 0 | 1 | 0 | 0 | 0 | 0 | 1 |
| **45** | ANQSFEYGAIGQCLPPCPMSH | 21 | 2 | 1 | 0 | 1 | 1 | 1 | 0 | 1 | 3 | 2 | 2 | 2 | 0 | 1 | 2 | 0 | 0 | 1 | 0 | 1 | -1 |
| **46** | ANSCDILTAIVWDSYMGTCFICNKH | 25 | 2 | 3 | 1 | 1 | 1 | 1 | 1 | 1 | 0 | 1 | 3 | 2 | 2 | 2 | 0 | 0 | 1 | 1 | 2 | 0 | -1 |
| **47** | SETNCGYPTKFCMVCATCEHRVVYTQCQ | 28 | 1 | 0 | 3 | 0 | 1 | 1 | 0 | 2 | 1 | 1 | 5 | 1 | 4 | 1 | 2 | 1 | 1 | 1 | 0 | 2 | 0 |
| **48** | GHCLTNSPLAGGCTGWLRI | 16 | 1 | 1 | 0 | 3 | 0 | 0 | 1 | 0 | 1 | 4 | 1 | 2 | 0 | 1 | 0 | 0 | 1 | 0 | 1 | 0 | 1 |
| **49** | NSDNKHCYYYTSGIYTRCDD | 20 | 0 | 1 | 0 | 0 | 0 | 0 | 0 | 4 | 0 | 1 | 2 | 2 | 2 | 2 | 0 | 1 | 1 | 1 | 3 | 0 | -1 |
| **5** | DITWDFVSTLVTACADRTMHCCKK | 24 | 2 | 1 | 2 | 1 | 1 | 1 | 1 | 0 | 0 | 0 | 3 | 1 | 4 | 0 | 0 | 1 | 2 | 1 | 3 | 0 | 0 |
| **51** | ALQSTIQSENCTFRGANCQL | 20 | 2 | 1 | 0 | 2 | 0 | 1 | 0 | 0 | 0 | 1 | 2 | 2 | 2 | 2 | 3 | 1 | 0 | 0 | 0 | 1 | 0 |
| **52** | KDGTCTKCVTECKGRYC | 17 | 0 | 0 | 1 | 0 | 0 | 0 | 0 | 1 | 0 | 2 | 4 | 0 | 3 | 0 | 0 | 1 | 3 | 0 | 1 | 1 | 2 |
| **53** | KKPKLEYSNTSLACGC | 16 | 1 | 0 | 0 | 2 | 0 | 0 | 0 | 1 | 1 | 1 | 2 | 2 | 1 | 1 | 0 | 0 | 3 | 0 | 0 | 1 | 2 |
| **SA15** | VTLKYKNWCYCTAL | 14 | 1 | 0 | 1 | 2 | 0 | 0 | 1 | 2 | 0 | 0 | 2 | 0 | 2 | 1 | 0 | 0 | 2 | 0 | 0 | 0 | 2 |
| **55** | **RGFSYLPQECVTDYLCG** | 17 | 0 | 0 | 1 | 2 | 0 | 1 | 0 | 2 | 1 | 2 | 2 | 1 | 1 | 0 | 1 | 1 | 0 | 0 | 1 | 1 | -1 |
| **SA6** | GCSFEYGKMGKDKCSR | 16 | 0 | 0 | 0 | 0 | 1 | 1 | 0 | 1 | 0 | 3 | 2 | 2 | 0 | 0 | 0 | 1 | 3 | 0 | 1 | 1 | 2 |
| **SA16** | **RIVCVSFGSLFIRRGKLS** | 18 | 0 | 2 | 2 | 2 | 0 | 2 | 0 | 0 | 0 | 2 | 1 | 3 | 0 | 0 | 0 | 3 | 1 | 0 | 0 | 0 | 4 |
| **SA11** | CDIELVSLCSKTHNVLC | 24 | 0 | 1 | 2 | 3 | 0 | 0 | 0 | 0 | 0 | 0 | 3 | 1 | 2 | 0 | 1 | 2 | 2 | 0 | 1 | 0 | -1 |
| **59** | **CYNWKSARDY** | 10 | 1 | 0 | 0 | 0 | 0 | 0 | 1 | 2 | 0 | 0 | 1 | 1 | 0 | 1 | 0 | 1 | 1 | 0 | 1 | 0 | 1 |
| **6** | FECSFPCKLSIGGPG | 15 | 0 | 1 | 0 | 1 | 0 | 2 | 0 | 0 | 2 | 3 | 2 | 2 | 0 | 0 | 0 | 0 | 1 | 0 | 0 | 1 | 0 |
| **61** | LGPKVSAGSLYCLCPG | 16 | 1 | 0 | 1 | 3 | 0 | 0 | 0 | 1 | 2 | 3 | 2 | 2 | 0 | 0 | 0 | 0 | 1 | 0 | 0 | 0 | 1 |
| **62** | TCFCFSVACRLDTGITFR | 18 | 1 | 1 | 1 | 1 | 0 | 3 | 0 | 0 | 0 | 1 | 3 | 1 | 3 | 0 | 0 | 2 | 0 | 0 | 1 | 0 | 1 |

| | | | | | | | | | | | | | | | | | | | | | | |
|---|---|---|---|---|---|---|---|---|---|---|---|---|---|---|---|---|---|---|---|---|---|---|
| **63** | WRCSVGPQ<br>TNKQNWTQA<br>LCCQKGQI | 25 | 1 | 1 | 1 | 1 | 0 | 0 | 2 | 0 | 1 | 2 | 3 | 1 | 2 | 2 | 5 | 1 | 2 | 0 | 0 | 0 | 3 |
| **SA7** | **YQSWDVWD<br>GVGYNC** | 14 | 0 | 0 | 2 | 0 | 0 | 0 | 2 | 2 | 0 | 2 | 1 | 1 | 0 | 1 | 1 | 0 | 0 | 0 | 2 | 0 | -2 |
| **65** | ADTLCCPVR<br>CGQTFSCIA | 18 | 2 | 1 | 1 | 1 | 0 | 1 | 0 | 0 | 1 | 1 | 4 | 1 | 2 | 0 | 1 | 1 | 0 | 0 | 1 | 0 | 0 |
| **66** | GAETACLTN<br>NTCFEAQAC<br>CYFTL | 23 | 4 | 0 | 0 | 2 | 0 | 2 | 0 | 1 | 0 | 1 | 4 | 0 | 4 | 2 | 1 | 0 | 0 | 0 | 0 | 2 | -2 |
| **67** | KDYVGRTLT<br>QGKCGC | 15 | 0 | 0 | 1 | 1 | 0 | 0 | 0 | 1 | 0 | 3 | 2 | 0 | 2 | 0 | 1 | 1 | 2 | 0 | 1 | 0 | 2 |
| **68** | NCQRCNHKS<br>ACSATYVPC<br>VVLTEI | 96 | 2 | 1 | 3 | 1 | 0 | 0 | 0 | 1 | 1 | 0 | 2 | 10 | 11 | 5 | 7 | 3 | 5 | 1 | 6 | 1 | 1 |
| **69** | ECIGGGRGR<br>CSFVSPQSE<br>RGGVGDEK | 26 | 0 | 1 | 2 | 0 | 0 | 1 | 0 | 0 | 1 | 7 | 2 | 3 | 0 | 0 | 1 | 3 | 1 | 0 | 1 | 3 | 0 |
| **70** | **CACFKKTEI<br>WFIR** | 13 | 1 | 2 | 0 | 0 | 0 | 2 | 1 | 0 | 0 | 0 | 2 | 0 | 1 | 0 | 0 | 1 | 2 | 0 | 0 | 1 | 2 |
| **71** | FGTCFIGRNT<br>TDGKVRITW<br>YWV | 22 | 0 | 2 | 2 | 0 | 0 | 2 | 2 | 1 | 0 | 3 | 1 | 0 | 4 | 1 | 0 | 2 | 1 | 0 | 1 | 0 | 2 |
| **72** | ACIDCFTRYG<br>TDEEVTLIDD | 20 | 1 | 2 | 1 | 1 | 0 | 1 | 0 | 1 | 0 | 1 | 2 | 0 | 3 | 0 | 0 | 1 | 0 | 0 | 4 | 2 | -5 |
| **SA1** | AGSANPQGE<br>DPWCTWDY<br>PICINHK | 24 | 2 | 2 | 0 | 0 | 0 | 0 | 2 | 1 | 3 | 2 | 2 | 1 | 1 | 2 | 1 | 0 | 1 | 1 | 2 | 1 | -2 |
| **74** | GHGVTFYNP<br>CSTLIMTLKC<br>CTKMLAVCP<br>K | 29 | 1 | 1 | 2 | 3 | 2 | 1 | 0 | 1 | 2 | 2 | 4 | 1 | 4 | 1 | 0 | 0 | 3 | 1 | 0 | 0 | 3 |
| **75** | **GQAKQSIRV<br>CRRTLKS** | 16 | 1 | 1 | 1 | 1 | 0 | 0 | 0 | 0 | 0 | 1 | 1 | 2 | 1 | 0 | 2 | 3 | 2 | 0 | 0 | 0 | 5 |
| **76** | ESCPTVPICG<br>NMNFGCCTS<br>TGV | 22 | 0 | 1 | 2 | 0 | 1 | 1 | 0 | 0 | 2 | 3 | 4 | 2 | 3 | 2 | 0 | 0 | 0 | 0 | 0 | 1 | -1 |
| **77** | QFGSYCMCH<br>HKTLCKNDS<br>VDL | 21 | 0 | 0 | 1 | 2 | 1 | 1 | 0 | 1 | 0 | 1 | 3 | 2 | 1 | 1 | 1 | 0 | 2 | 2 | 2 | 0 | 0 |
| **78** | ACPSTFTIDA<br>CAATGCNPK<br>DL | 22 | 4 | 1 | 0 | 1 | 0 | 1 | 0 | 0 | 2 | 1 | 2 | 2 | 0 | 0 | 1 | 2 | 2 | 0 | 1 | 0 | -1 |
| **SA3** | WCGNACTN<br>WKWSNGAG<br>TGC | 19 | 2 | 0 | 0 | 0 | 0 | 0 | 3 | 0 | 0 | 4 | 3 | 1 | 2 | 3 | 0 | 0 | 1 | 0 | 0 | 0 | 1 |
| | **Total of AA's** | **178<br>3** | **102** | **75** | **80** | **94** | **18** | **56** | **57** | **66** | **86** | **178** | **178** | **131** | **149** | **82** | **55** | **66** | **87** | **25** | **89** | **59** | **Charge[c]<br>%** |

| | | | | | | | | | | | | | | | | | | | | | | | | |
|---|---|---|---|---|---|---|---|---|---|---|---|---|---|---|---|---|---|---|---|---|---|---|---|---|
| | Percentage % | | 5,9 | 4,3 | 4,6 | 5,4 | 1,0 | 3,2 | 3,3 | 3,8 | 4,9 | 10 | 10 | 7,6 | 8,6 | 4,7 | 3,2 | 3,8 | 5,0 | 1,5 | 5,1 | 3,4 | - | 43 |
| | **Aminoacid presence in core peptides**[b] | | **55** | **50** | **48** | **49** | **16** | **42** | **36** | **45** | **43** | **64** | 76 | **66** | **59** | **50** | **30** | **40** | **cor rec t or not** | **23** | **50** | **40** | + | 37 |
| | Percentage % | | 70 | 63 | 61 | 62 | 20 | 54 | 46 | 57 | 54 | 81 | 96 | 84 | 75 | 63 | 38 | 51 | 65 | 29 | 63 | 51 | N[d] | 20 |
| | | | **A** | **I** | **V** | **L** | **M** | **F** | **W** | **Y** | **P** | **G** | **C** | **S** | **T** | **N** | **Q** | **R** | **K** | **H** | **D** | **E** | | |

[a] Total aminoacid per core sequence

[b] Count of times an amino acid is observed in SyncA's core.

[c] Summary of net charge[2] found in SyncA's. Percentage

[d] Neutral charge (N)

**Supplementary Table S2**. Summary of plasmids and strains used in this study. All the used plasmids were cloned into *L. lactis* NZ9000.

| Strain | Mutations | Characteristic | References |
|---|---|---|---|
| *Micrococcus flavus* | | Sensitive strain | [3] |
| *Lactococus lactis* NZ9000 | *pepN:nisRK* | Nisin inducible strain used in cloning and peptides expression | [4] |
| pNZ-nisP, pIL253 | | NisP soluble producer strain, Cm$^R$ , Ery$^R$ | [3] |
| pNZe-NisP8H | | Ery$^{R,}$ Cm$^{R.}$ NisP producer strain | [3] |
| pTLR-SyncM | | SyncM lanthipeptide synthase cloned under nisin inducible promoter ($P_{nis}$ promoter), Ery$^R$ | [6] |
| pNZ8048 | | Nisin inducible expression vector, Cm$^R$, | [5] |
| pNZ8048-SyncA2 | | SyncA2 cloned under $P_{nis}$ promoter in pNZ8048 Cm$^R$ | [6] |
| pNZ8048-SA2 -S12A | S12A | SA2 -S12A cloned under $P_{nis}$ promoter, Cm$^R$ | |
| pNZ8048-SA2-SWAP | Ring(C2S/S12C) | SA2-SWAP cloned under $P_{nis}$ promoter, Cm$^R$ | This work |
| pNZ8048-SA2-+1A | +1A (P4_F5insA) | under $P_{nis}$ promoter, Cm$^R$ | This work |

| | | | |
|---|---|---|---|
| pNZ8048- SA2-+2A | +2A (P4_F5insAA) | under $P_{nis}$ promoter, $Cm^R$ | This work |
| pNZ8048- SA2-+3A | +3A (P4_F5insAAA) | under $P_{nis}$ promoter, $Cm^R$ | This work |
| pNZ8048- SA2-+4A | +4A (P4_F5insAAAA) | under $P_{nis}$ promoter, $Cm^R$ | This work |
| pNZ8048-SyncA6 | | SyncA6 cloned under under $P_{nis}$ promoter, $Cm^R$ | [5] |
| pNZ8048-SyncA6- SA6-SC | Ring1(C2S/S15C)-Ring2( S3C /C14S) | SA6-SC cloned under $P_{nis}$ promoter, $Cm^R$ | This work |
| pNZ8048-SyncA6- SA6-AS | ΔRing1(C2A/S15A) | SA6-AS cloned under $P_{nis}$ promoter, $Cm^R$ | This work |
| pNZ8048-SyncA6- SA6-AC | ΔRing1 (C2A/S15A)-Ring2(S2C /C14S) | SA6-AC cloned under $P_{nis}$ promoter $Cm^R$ | This work |
| pNZ8048-SyncA6- SA6-CA | ΔRing2(S2A/C14S) | SA6-CA cloned under $P_{nis}$ promoter $Cm^R$ | This work |
| pNZ8048-SyncA6- SA6-SA | Ring1(C2S/S15) -ΔRing2(S2A/C14S) | SA6-SA cloned under $P_{nis}$ promoter $Cm^R$ | This work |
| pNZ8048-SyncA2LE- SA2.1 | F5K | SA2.1 cloned after SA2- LEADER -ASPR under $P_{nis}$ promoter, $Cm^R$ | This work |
| pNZ8048-SyncA2LE- SA2.2 | F5K_D9K | SA2.2 cloned after SA2- LEADER -ASPR under $P_{nis}$ promoter, $Cm^R$ | This work |
| pNZ8048-SyncA2LE- SA-2.3 | F5W_Y8W_D9R_L13R_L14R-ΔS15-ΔP16 | SA-2.3 cloned after SA2-LEADER-ASPR under $P_{nis}$ promoter, $Cm^R$ | This work |
| pNZ8048-SyncA2LE- SA-2.4 | Most of the structure change | SA-2.4 cloned after SA2- LEADER -ASPR pNZ8048, under $P_{nis}$ promoter, $Cm^R$ | This work |
| pNZ8048-SyncA2LE- SA-2.5 | Most of the structure change | SA-2.5 cloned after SA2-LEADER ASPR pNZ8048, under $P_{nis}$ promoter, $Cm^R$ | This work |
| pNZ8048-SyncA2LE- SAUW.1 | Q1R_N7R | SAUW.1cloned after SA2- LEADER -ASPR under $P_{nis}$ promoter, $Cm^R$ | This work |
| pNZ8048-LACM | insS15[7] | LACM cloned after SA2- LEADER -ASPR pNZ8048, under $P_{nis}$ promoter, $Cm^R$ $Ery^R$, *sync*M cloned in pTLR under $P_{nis}$ promoter | This work |

| | | | |
|---|---|---|---|
| pNZ8048- LACPINM | S16C[7] | LACPINM cloned after SA2- LEADER -ASPR pNZ8048, under $P_{nis}$ promoter, $Cm^R$ | This work |
| pNZ8048- -BACM | C3S[8] | BACM cloned after SA2- LEADER -ASPR pNZ8048, under $P_{nis}$ promoter, $Cm^R$ | This work |
| pNZ8048 - SUB3M | insS3_insC14[9] | SUB3M cloned after SA2- LEADER -ASPR pNZ8048, under $P_{nis}$ promoter, $Cm^R$ | This work |
| pNZ8048 - M1M | Murepavadin mimic[1] | M1M cloned after SA2- LEADER -ASPR pNZ8048, under $P_{nis}$ promoter, $Cm^R$ | This work |
| pNZ8048 - M2M | Murepavadin mimic | M2M cloned after SA2- LEADER -ASPR pNZ8048, under $P_{nis}$ promoter, $Cm^R$ | This work |
| pNZ8048-SyncA7 | | under $P_{nis}$ promoter $Cm^R$ | [6] |
| pNZ8048-SyncA7LE-SyncA7.1 | D5K_D8K | Desired core peptide was cloned after SA7-LEADER GG pNZ8048, under $P_{nis}$ promoter, $Cm^R$ $Ery^R$, *sync*M cloned in pTLR under $P_{nis}$ promoter | This work |
| pNZ8048-SyncA7LE-SyncA7.2 | -Y1R_G2R_D5R_D8R_Y12R | Desired core peptide was cloned after SA7-LE LEADER GG pNZ8048, under $P_{nis}$ promoter, $Cm^R$ $Ery^R$, *sync*M cloned in pTLR under $P_{nis}$ promoter | This work |
| pNZ8048-SyncA2LE- SA2.2, pTLR-SyncM | F5K_D9K | Desired core peptide was cloned after SA2-LEADER -ASPR pNZ8048, under $P_{nis}$ promoter, $Cm^R$ $Ery^R$, *sync*M cloned in pTLR under $P_{nis}$ promoter | This work |
| pNZ8048-SyncA2LE- SA-2.3, pTLR-SyncM | F5W_Y8W_D9R_L13R_L14R-ΔS15-ΔP16 | Desired core peptide was cloned after SA2-LEADER-ASPR pNZ8048, under $P_{nis}$ promoter, $Cm^R$ $Ery^R$, *sync*M cloned in pTLR under $P_{nis}$ promoter | This work |
| pNZ8048-SyncA2LE- SA-2.4, pTLR-SyncM | Most of the structure change | Desired core peptide was cloned after SA2-LEADER -ASPR pNZ8048, under $P_{nis}$ promoter, $Cm^R$ $Ery^R$, *sync*M cloned in pTLR under $P_{nis}$ promoter | This work |

| | | | |
|---|---|---|---|
| pNZ8048-SyncA2LE- SA-2.5, pTLR-SyncM | Most of the structure change | Desired core peptide was cloned after SA2-LEADER ASPR pNZ8048, under $P_{nis}$ promoter, $Cm^R$ $Ery^R$, *sync*M cloned in pTLR under $P_{nis}$ promoter | This work |
| pNZ8048-SyncA2LE-SAUW.1, pTLR-SyncM | -Q1R_N7R | Desired core peptide was cloned after SA2-LEADER -ASPR pNZ8048, under $P_{nis}$ promoter, $Cm^R$ $Ery^R$, *sync*M cloned in pTLR under $P_{nis}$ promoter | This work |
| pNZ8048-LACM_, pTLR-SyncM | _+S15[7] | Desired core peptide was cloned after SA2-LEADER -ASPR pNZ8048, under $P_{nis}$ promoter, $Cm^R$ $Ery^R$, *sync*M cloned in pTLR under $P_{nis}$ promoter | This work |
| pNZ8048- LACPINM, pTLR-SyncM | S16C[7] | Desired core peptide was cloned after SA2-LEADER -ASPR pNZ8048, under $P_{nis}$ promoter, $Cm^R$ $Ery^R$, *sync*M cloned in pTLR under $P_{nis}$ promoter | This work |
| pNZ8048- -BACM, pTLR-SyncM | C3S [8] | Desired core peptide was cloned after SA2-LEADER -ASPR pNZ8048, under $P_{nis}$ promoter, $Cm^R$ $Ery^R$, *sync*M cloned in pTLR under $P_{nis}$ promoter | This work |
| pNZ8048-SUB3M, pTLR-SyncM | +S3+C14[9] | Desired core peptide was cloned after SA2-LEADER -ASPR pNZ8048, under $P_{nis}$ promoter, $Cm^R$ $Ery^R$, *sync*M cloned in pTLR under $P_{nis}$ promoter | This work |
| pNZ8048- M1M, pTLR-SyncM | Murepavadin mimic[1] | Desired core peptide was cloned after SA2-LEADER -ASPR pNZ8048, under $P_{nis}$ promoter, $Cm^R$ $Ery^R$, *sync*M cloned in pTLR under $P_{nis}$ promoter | This work |
| pNZ8048 - M2M, pTLR-SyncM | Murepavadin mimic | Desired core peptide was cloned after SA2-LEADER -ASPR pNZ8048, under $P_{nis}$ promoter, $Cm^R$ $Ery^R$, *sync*M cloned in pTLR under $P_{nis}$ promoter | This work |

| | | | |
|---|---|---|---|
| pNZ8048-SyncA7 | | under $P_{nis}$ promoter $Cm^R$ | [6] |
| pNZ8048-SyncA7LE-SyncA7.1 | -D5K_D8K | Desired core peptide was cloned after SA7-LEADER GG<br>pNZ8048, under $P_{nis}$ promoter, $Cm^R$<br>$Ery^R$, *sync*M cloned in pTLR under $P_{nis}$ promoter | This work |
| pNZ8048-SyncA7LE-SyncA7.2 | -Y1R_G2R_D5R_D8R_Y12R | Desired core peptide was cloned after SA7-LE LEADER GG<br>pNZ8048, under $P_{nis}$ promoter, $Cm^R$<br>$Ery^R$, *sync*M cloned in pTLR under $P_{nis}$ promoter | This work |

## Reference

(1) McLaughlin, M. I., and van der Donk, W. A. (2020) The Fellowship of the Rings: Macrocyclic Antibiotic Peptides Reveal an Anti-Gram-Negative Target, *Biochemistry 59*, 343-345.

(2) Wang, G., Li, X., and Wang, Z. (2016) APD3: the antimicrobial peptide database as a tool for research and education, *Nucleic Acids Res 44*, D1087-1093.

(3) Montalban-Lopez, M., Deng, J., van Heel, A. J., and Kuipers, O. P. (2018) Specificity and Application of the Lantibiotic Protease NisP, *Front Microbiol 9*, 160.

(4) Kuipers, O. P., de Ruyter, P. G., Kleerebezem, M., and de Vos, W. M. (1997) Controlled overproduction of proteins by lactic acid bacteria, *Trends Biotechnol 15*, 135-140.

(5) Kuipers, O. P., de Ruyter, P. G. G. A., Kleerebezem, M., and de Vos, W. M. (1998) Quorum sensing-controlled gene expression in lactic acid bacteria, *Journal of Biotechnology 64*, 15-21.

(6) Arias-Orozco, P., Inklaar, M., Lanooij, J., Cebrian, R., and Kuipers, O. P. (2021) Functional Expression and Characterization of the Highly Promiscuous Lanthipeptide Synthetase SyncM, Enabling the Production of Lanthipeptides with a Broad Range of Ring Topologies, *ACS Synth Biol 10*, 2579-2591.

(7) van der Kraan, M. I. A., Groenink, J., Nazmi, K., Veerman, E. C. I., Bolscher, J. G. M., and Nieuw Amerongen, A. V. (2004) Lactoferrampin: a novel antimicrobial peptide in the N1-domain of bovine lactoferrin, *Peptides 25*, 177-183.

(8) Wu, M., and Hancock, R. E. W. (1999) Improved Derivatives of Bactenecin, a Cyclic Dodecameric Antimicrobial Cationic Peptide, *Antimicrobial Agents and Chemotherapy 43*, 1274-1276.

(9) Hilpert, K., Volkmer-Engert, R., Walter, T., and Hancock, R. E. (2005) High-throughput generation of small antibacterial peptides with improved activity, *Nat Biotechnol 23*, 1008-1012.
